# Rescue Radiosensitization of Pancreatic Cancer via PD-L1/TGF-β1 Dual-Blockade Nanotherapy as Evaluated in 3-Dimensional Microtumors

**DOI:** 10.34133/bmr.0335

**Published:** 2026-03-04

**Authors:** Di Chen, Lei He, Liang Chen, Chen Ye, Fei Duan, Xiaofei Zhu, Wei Jing, Huojun Zhang, Wei Li

**Affiliations:** ^1^ Department of Nanomedicine, Translational Medicine Research Center, & Shanghai Key Laboratory of Nautical Medicine and Translation of Drugs and Medical Devices, Naval Medical University, Shanghai 200433, China.; ^2^ Department of Radiation Oncology, Changhai Hospital affiliated to Naval Medical University, Shanghai 200433, China.; ^3^ Department of Radiation Oncology, Huadong Hospital, Fudan University, Shanghai 200040, China.; ^4^ Department of Hepatobiliary and Pancreatic Surgery, Changhai Hospital affiliated to Naval Medical University, Shanghai 200433, China.

## Abstract

Radiation-induced immunological and stromal changes in the pancreatic tumor microenvironment (TME) often develop adaptive radioresistance in clinical. Among these changes, cellular compensatory programmed cell death-ligand 1 (PD-L1) overexpression induced by radiation will promote the adaptive immune evasion, limiting the radiation-mediated antitumor effect. Regrettably, the PD-L1 overexpression will be further potentiated by transforming growth factor-β1 (TGF-β1) that abundantly secreted by irradiated pancreatic stellate cells. This further fosters an immunosuppressive TME, which constitutes one of the key factors contributing to the limited efficacy of combining radiotherapy with programmed cell death protein 1 (PD-1)/PD-L1 blockade in pancreatic ductal adenocarcinoma. To counteract this resistance mechanism, we developed a TME-responsive nanogel (pirfenidone@nanogel–hyaluronidase–anti-PD-L1 [PFD@NGHP]) for rescuing radiosensitization. The PFD@NGHP is composed of a reduction-sensitive core encapsulating pirfenidone and a cationic surface corona of hyaluronidase and anti-PD-L1 antibodies. At the intercellular level, PFD@NGHP effectively inhibited TGF-β1 secretion by about 50% and targeted PD-L1 for antibody-dependent cell-mediated cytotoxicity. In the 3-dimensional stromal microtumors, PFD@NGHP effectively penetrated in stroma (>400 μm in depth), suppressed pancreatic stellate cells, and potentiated radiosensitization. In murine models, PFD@NGHP ameliorated the stroma through TGF-β1 inhibition, subsequently increased T cell infiltration of about 30% CD8^+^ T cells, and amplified the efficacy of PD-L1 blockade. This effect synergized radiotherapy to sustain tumor regression and generate abscopal effects. Collectively, our study demonstrates that PFD@NGHP targets the TGF-β1–PD-L1 axis in a cascading manner, offering a promising clinical strategy to overcome the adaptive radioresistance of irradiated pancreatic ductal adenocarcinoma while providing a potential platform for translational nanomedicine evaluation.

## Introduction

Pancreatic ductal adenocarcinoma (PDAC) is still the highly lethal malignancy with a dismal 5-year survival rate of 13% in the United States [1,2]. Although treatment of PDAC with optimal surgery, radiotherapy, immune-based therapies, and targeted approaches has advanced, survival improvements have been real yet modest. Tumor recurrence and metastatic progression continue to present significant clinical challenges in the surgery and chemotherapy of PDAC [2,3]. Immunotherapies still demonstrate limited efficacy, necessitating synergistic integration with conventional treatments [4,5]. Breakthrough targeted therapies, such as KRAS and poly(adenosine diphosphate-ribose) polymerase inhibitors, are transformative for specific genetic subsets, while remaining clinically restricted by mutational limitations [2,3]. Radiotherapy has emerged as a cornerstone treatment for PDAC, offering a therapeutic alternative given that curative resection is only feasible in fewer than 20% of patients [3]. Especially, stereotactic body radiotherapy (SBRT) has inspiring advantages in achieving local control, priming antitumor immunity, and improving the quality of life [6]. However, the 1-year local control rate for patients with PDAC treated with SBRT still remains below 80%, meaning that many patients do not benefit from radiotherapy [7,8]. Increasing evidence highlights the crucial role of the immunosuppressive environment postradiotherapy in PDAC for hindering the effect of radiation-induced immunogenic cell death (ICD), preventing tumor regression after treatment [9,10]. Radiotherapy has attracted considerable attention owing to its well-known potential for immunostimulatory effects. After radiotherapy, inflammatory signals are triggered through the activation of cell survival pathways and stimulation of the innate immune system, which will promote immune cells recruitment [9,11]. Radiation promotes the up-regulation of major histocompatibility complex (MHC) molecules and presentation of novel antigenic determinants on the surface of cancer cells, thereby rendering them more susceptible to recognition by T cells [11]. The radiation-induced dead cells will generate damage-associated molecular patterns (DAMPs), which subsequently induce the ICD [10]. This response changes the predominant tumor microenvironment (TME) cytokine profile toward an immunostimulatory profile. However, the survival cancer cells will up-regulate the expression of programmed cell death-ligand 1 (PD-L1) through multiple mechanisms to evade ICD [8,11]. The activation of DNA damage signaling pathways, such as ATM-ATR/Chk1 and the cGAS/STING pathway, contributes to the up-regulation of PD-L1 expression [12,13]. In addition, immunostimulatory cytokines such as interferon-γ, produced during antitumor immunity, further enhance the PD-L1 expression [14]. This high PD-L1 expression in tumor tissue indicates a higher tumor proportion score, which correlates with increased sensitivity to immunotherapy [8,15]. Therefore, the anti-programmed cell death protein 1 (PD-1)/PD-L1 treatments have been widely used as a combination partner for radiotherapy in clinical management of many malignancies.

Nevertheless, the notorious PDAC stroma, composed of dense hyaluronan-rich extracellular matrix (ECM) and pancreatic stellate cells (PSCs), maintains complex communication with malignant tumor cells to promote the cancer stem cell phenotype, epithelial-to-mesenchymal transition (EMT), and immunosuppression [16,17]. It is currently believed that transforming growth factor-β1 (TGF-β1) secreted from stroma is another stimulus to up-regulate the PD-L1 expression of cancer cells [14,18]. Although TGF-β1, as a key member of the TGF-β family, plays a critical role in tumor proliferation and EMT, contributing to radiotherapy resistance, numerous studies have elucidated the mechanism by which TGF-β1 induces PD-L1 overexpression on cancer cells inducing immune escape [18,19]. For instance, the canonical TGF-β1/SMAD pathway and the TGF-β1/EMT pathway have been shown to up-regulate PD-L1 expression in colorectal cancer, non-small-cell lung cancer, and breast cancer [20,21]. In PDAC, TGF-β1 is recognized as a key factor driving PD-L1 expression by strengthening the pyruvate kinase M2 (PKM2)–signal transducers and activators of transcription 1 (STAT1) interaction. Under TGF-β1 stimulation, PKM2 nuclear translocation and STAT1 phosphorylation are enhanced, facilitating the transactivation of PD-L1 by the binding of PKM2 and STAT1 to its promoter, thereby up-regulating PD-L1 expression [22]. PSCs, excluding certain immune cells, are the main producers of TGF-β1 in pancreatic cancer, promoting the development of desmoplasia [16,23]. In addition, reactive oxygen species (ROS) accumulated in irradiated PSCs can induce the hypersecretion of TGF-β1 via activation of multiple signaling pathways, such as the canonical SMAD pathway or noncanonical pathways [24,25]. This feedback secretion of TGF-β1 can promote the immunosuppression environment and highly dense fibrotic stroma, impairing the adaptive immune priming by radiotherapy and the subsequent tumor regression. Therefore, the radiation-induced TGF-β1 hypersecretion will promote the cancer cells’ ability to counteract immune cytotoxicity, which finally induced adaptive resistance. Due to the correlation between TGF-β1 and PD-L1, which jointly mediate resistance mechanisms within the irradiated TME, the simultaneous inhibition of both pathways may allow for increased overall efficacy of radiotherapy [18,26]. Moreover, the dual inhibition of TGF-β1 and PD-L1 is now intensely investigated in a lot of clinical trials including NCT02937272, NCT04551950, etc. [26].

In dual inhibition treatment, TGF-β-targeting therapies have matured through various methods, including small-molecule inhibitors and antibodies [27]. Pirfenidone (PFD) is a pyridone compound with antifibrotic effect via TGF-β inhibition, which is approved for treating idiopathic pulmonary fibrosis [23]. As revealed in many investigations, PFD impeded the PSC/cancer-associated fibroblast proliferation, TGF-β1 production, and collagen synthesis, which mitigated stromal desmoplasia [23]. For instance, PFD may alter the pancreatic milieu and alleviate fibrosis through the regulation of tumor–stroma interactions via the transglutaminase 2 (TGM2)/nuclear factor κB(NF-κB)/platelet-derivedgrowth factor B (PDGFB) pathway [28]. However, PFD is still subject to dosage limitations, delivery efficacy, and perfusion volume, especially in the stiff PDAC [29,30]. In addition, the dual inhibition by 2 molecules may not provide the localized and simultaneous optimal suppression in irradiated TME comparing with single-molecule therapy targeting both pathways, as evidenced by the superior preclinical activity of bintrafusp alfa [31]. Bintrafusp alfa, a bifunctional fusion protein, was reported to simultaneously inhibit TGF-β and PD-L1 to synergize radiotherapy and eradicate therapy-resistant tumors with poor immune infiltration. In addition, Tapia-Galisteo et al. [32] described another bispecific antibody, AxF (scFv)2, designed for dual blockade of PD-L1 and TGF-β, based on the premise that combining it with T cell redirecting strategies would overcome TME-mediated immunosuppression and enhance clinical benefit. However, these molecules are still subject to inadequate cellular suppression, intratumoral dosage limitations, and patient exclusions, and determining the optimal balance between efficacy and toxicity remains a challenge.

Incorporation of TGF-β- and PD-L1-targeting agents into nanoparticle formulations could effectively improve the above shortcomings. The stimuli-responsive modifications including hyperthermic (>37 °C), acidic (pH < 6), or reductive environment and targeting modifications including antibodies, enzymes, or peptides have nanoparticles tailored to enhance pharmacokinetic characteristics and selective accumulation in tumors [33,34]. Therefore, these nanomedicines could generate controlled release of payloads with a selective spatial and temporal approach to drastically reducing the dose administration and the additional toxic risk [35]. We have previously reported an intelligent nanogel system “GEM@NGH” that ablated the stromal hyaluronan by hyaluronidase (HAase) to enhance the gemcitabine intratumoral delivery corresponding to significant regression in pancreatic cancer [34]. In addition, in our previous work [34], an in vitro 3-dimensional (3D) tumor model constructed for simply mimicking TME of PDAC was important for the development of biomimetic testing platforms to advance the preclinical evaluation of nanomedicines.

**Fig. 1.**
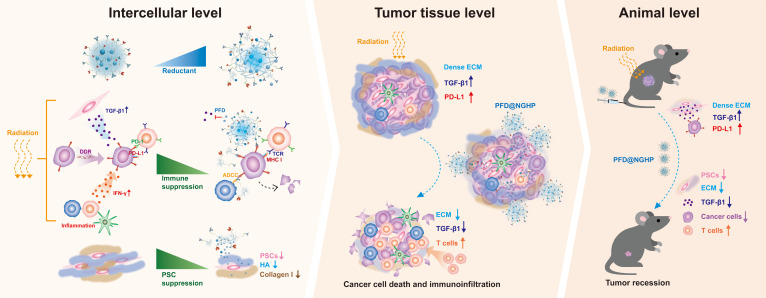
The mechanism that PFD@NGHP infiltrated into tumor tissues and disintegrated in the reductive environment to release PFD, inhibiting TGF-β1 and blocking PD-L1 to rescue immunosuppression, thereby leading to robust tumor regression. DDR, DNA damage response; IFN-γ, interferon-γ; TCR, T cell receptor; MHC I, major histocompatibility complex I.

Thus, to examine the effect of dual inhibition in sensitizing PDAC to radiotherapy, we reconstructed in vitro cocultured 3D microtumors using murine cell lines and human cell lines, respectively, to recapitulate PDAC stromal spatial bioarchitecture. Moreover, an orthotopic tumor model transplanted in culture Matrigel was also applied for in vitro evaluation of intratumoral penetration. As referenced in our previous work [34], we prepared a reduction-sensitive nanogel (PFD@NGHP) consisting of the payloads of PFD and the coronal of HAase and anti-PD-L1 antibodies (αPD-L1) arrayed on the cationic surface. Its physical and chemical properties were assayed by dynamic and static light scattering (DLS/SLS) and enzyme-linked immunosorbent assay (ELISA). PFD@NGHP has access to intratumor via HAase ablating hyaluronan. In addition, it exhibited redox-sensitive disintegration specifically triggered by stromal reductants. Therefore, the released PFD inhibited the proliferation of PSCs and function of TGF-β1, while αPD-L1 suppressed immune evasion. In this work, simultaneously blocking the TGF-β1 and PD-L1 pathways with PFD@NGHP would synergize with radiotherapy to improve the therapeutic efficacy of PDAC and would help to provide a meaningful strategy to treat other desmoplastic tumors (Fig. 1).

## Materials and Methods

### Materials and cell lines

Branched polyethylenimines (bPEIs; 25 kD), 2-iminothiolane (2-IT) HCl, dithiobis(succinimidyl propionate) (DSP), Rhodamine (Rho) B isothiocyanate, l-glycine, and fluorescein isothiocyanate (FITC) were all purchased from Merck. Maleimide–polyethylene glycol–succinimidyl carboxymethyl ester (mPEGS; 3,500 kD) was purchased from Creative PEGWorks (USA). Dimethyl sulfoxide (DMSO) was purchased from J&K Scientific (China) and freshly dried before use. Amplite fluorimetric maleimide assay kit was purchased form AAT Bioquest (Sunnyvale, CA). TNBS (2,4,6-trinitrobenzenesulfonic acid) assay kit was obtained from Tokyo Chemical Industry (catalog no. T2024). The reduced glutathione (GSH) assay kit and Ellman’s reagent were obtained from Beyotime (China). The CytoTox 96 nonradioactive cytotoxicity assay was purchased form Promega (USA). Cell Counting Kit-8 (CCK-8) was purchased from DOJINDO (Japan). Hyaluronan, HAase (type IV-S), and Matrigel (matrix for organoid culture) were all purchased from Merck. Collagen I was purchased from BD (USA). PFD was purchased from Selleck. Fetal bovine serum (FBS), phosphate-buffered saline (PBS), Dulbecco’s modified Eagle’s medium (DMEM), and F12/DMEM were products of Gibco (NY, USA). PANC-1 cells, PAN02 cells, human PSCs (hPSCs), and mouse PSCs (mPSCs) were generously provided from Gastroenterology Department of Changhai Hospital, the Naval Medical University. hTERT-HPNE cells (normal human pancreatic ductal epithelial cells) were purchased from Shanghai Institute of Biochemistry and Cell Biology, Chinese Academy of Sciences. The following antibodies were used for synthesis, Western blotting, immunochemistry, and immunofluorescence: avelumab (αPD-L1) (MedChemExpress, catalog no. HY-108730), α-tubulin antibody (Cell Signaling Technology, catalog no. 2125), α-smooth muscle actin (α-SMA) antibody (Abcam, catalog no. ab265588), anti-TGF-β1 antibody (Abcam, catalog no. ab215715), anti-collagen I antibody (Abcam, catalog no. ab138492), Alexa Fluor 594 goat anti-rabbit immunoglobulin G (IgG) (H+L) cross-adsorbed secondary antibody (Thermo Fisher Scientific, catalog no. A-11012), and 4′,6-diamidino-2-phenylindole (DAPI; Beyotime, China, catalog no. C1006). Biotinylated hyaluronic acid (HA) binding protein was purchased form Merck (catalog no. 385911). Cyanine 3 tyramide signal amplification (CY3-TSA), CY5-TSA, and FITC-TSA were all purchased form Servicebio (China). Cancer cells were grown in DMEM, and PSCs were grown in F12/DMEM, which were both supplemented with 10% FBS and 1% penicillin–streptomycin in a humidified environment with 5% CO_2_ at 37 °C. The apparatus mainly included ALV/CGS-3 DLLS (ALV/Laser, Germany), ultraviolet (UV)-visible spectrophotometer (Varian, USA), and ultrapure water (18.2 MΩ/cm) produced by the Millipore Milli-Q Plus Water Purification System from Millipore, transmission electron microscope (TEM; H-7000 Electron Microscope, Hitachi), flow cytometer of Attune NxT (Invitrogen, USA), and confocal laser scanning microscope (CLSM; Zeiss LSM 980, Carl Zeiss Meditec AG, Jena, Germany).

### Synthesis of nanogel

The reactions were implemented in the following steps similar to our previous work [34]. The DSP and bPEI were respectively dissolved in anhydrous DMSO under nitrogen atmosphere. The filtered DSP/DMSO was slowly injected into a bPEI (25 kD)/DMSO solution with a molar ratio of 1:1 (DSP:PEI) at a rate of 0.4 ml/h by a programmable syringe pump (KD Scientific). After 2 h of vigorous stirring under nitrogen atmosphere at room temperature, the reaction mixture was quenched with 0.5 ml of deionized water and subsequently purified via dialysis (molecular weight cutoff: 100-kD membrane) against deionized water. The resultant nanogel was characterized by DLS/laser light scattering (LLS) to determine its physicochemical properties.

TNBS assay was then performed according to the described method to calculate the amount of residual primary amines in naked PEI nanogels [36]. The finally obtained PEI nanogels after dialysis and the unreacted PEI removed during dialysis were both collected and stored for subsequent detection. TNBS reagent (25 μl) was added to 0.5 ml of each standard, and sample solutions were diluted in 0.1 M borate buffer (pH 9.5). After 45 min of incubation at 25 °C, the absorbance of the reaction mixtures was measured at 420 nm using a microplate reader (BIOBASE, China).

Then, the nanogel was reacting with the mPEGS solution (5 mg/ml) at a mass ratio of 1:27 (PEI:mPEGS) in PBS with nitrogen protection for 2 d at room temperature to obtain the complete modification. The modified nanogel was finally dialyzed to remove the unreacted mPEGS. Finally, the maleimides modified on nanogels were monitored by the Amplite Rapid Colorimetric Maleimide Quantitation Kit.

### Preparation of nanogel–HAase–αPD-L1 intelligent system

As is known, 2-IT serves as a versatile reagent for specifically modifying primary amines (–NH_2_) through the targeted introduction of sulfhydryl (–SH) moieties. HAase and antibodies were functionalized with 2-IT to introduce sulfhydryl groups for subsequent mPEGS conjugation onto the nanogel. For HAase modification, the enzyme was dissolved in PBS (pH 8.0; containing 5 mM EDTA) at a concentration of 1 mg/ml. A 100-fold molar excess of aqueous 2-IT solution relative to HAase was then introduced under nitrogen protection with maintained 5 mM EDTA concentration. The reaction proceeded for 1 h at room temperature. The resulting thiolated enzymes were subsequently purified through dialysis against PBS (pH 8.0; 5 mM EDTA) under nitrogen protection. Thiolated antibodies were also prepared by the same method. The sulfhydrylation efficiency can be determined by Ellman assay. Finally, the mPEGS-modified nanogel was immediately mixed with sulfhydryl-activated enzymes/antibodies with a molar ratio of 1:2:2 (maleimides:HAase:αPD-L1). The solution was gently stirred overnight at 4 °C. The final NGHP was obtained after dialysis using a membrane with a 300-kD molecular weight cutoff in deionized water.

### Physicochemical property characterization of NGHP and PFD-loaded NGHP

The physicochemical characterization of NGHP was conducted via DLS/SLS analysis, determining key macromolecular parameters including *M_w_*, *R_h_*, *R_g_*, and *ρ*(*R_g_/R_h_*). In the SLS assay, the dn/dc of nanogel was about 0.279 ± 0.004 ml/g [34,37]. The synthesized products were first freeze-dried in a vacuum before quantification. Then, the quantified samples were diluted to different concentrations with 10 mM NaCl solution to be further tested. For testing in vivo stability of NGHP, the DMEM containing 10% FBS was used to mimic serum, and NGHP was mixed with the DMEM for 0 to 96 h before the DLS assay. Zeta potential measurements were conducted in deionized water using Zetasizer Nano ZS instrument (Malvern Instruments, UK). TEM was used to observe the morphology of nanoparticles without staining.

PFD aqueous solution (4 mg/ml) was added into the NGHP solution with a mass ratio of 4:1 (PFD:NGHP) and gently stirred overnight at room temperature in N_2_. Then, the mixture was dialyzed in PBS to remove the remaining PFD. Finally, PFD@NGHP was obtained and assayed by UV spectrophotometer to determine the final content of PFD.

### Reductive activity assay of extracellular medium

Extracellular thiols were quantified using Ellman’s reagent according to the manufacturer’s instructions. Briefly, cells (PANC-1, SW1990, PAN02, hTERT-HPNE, hPSC, and mPSC) were cultured or cocultured (cancer cell:PSC = 1:2) in conditioned media at 37 °C with 5% CO_2_. When cells reached approximately 90% confluence in 6-well plates, culture medium were replaced by fresh medium, with a subsequent incubation of 6 h. Then, 500 μl of medium of different cells were centrifuged at 10,000 rpm for 2 min at 4 °C. The supernatants were diluted with reaction buffer (100 mM sodium phosphate [pH 8.0] containing 1 mM EDTA) and then mixed with 50 μl of Ellman’s reagent solution. After incubation at room temperature for 15 min, 200 μl of reaction mixture was transferred to a 96-well plate and was measured photometrically at 412 nm. The concentration of thiols was calculated according to the GSH calibration curve.

### Stabilization of nanogel in reductive environment

To evaluate the reductive responsiveness of nanogels, nanogels (0.5 mg/ml) were dispersed in PBS solutions containing varying concentrations of GSH (0 μM, 50 μM, 200 μM, 500 μM, 1 mM, and 5 mM) and incubated at 37 °C for different durations. The size distributions of these samples were measured by DLS after 12, 24, and 48 h of incubation.

### Drug encapsulation efficiency and in vitro release profile

The UV-visible spectrophotometer was used to assay the PFD encapsulation efficiency (EE) and encapsulation content (EC) of the NGHP. PFD standard solutions were prepared with deionized water at concentrations of 5, 10, 25, 50, 100, and 500 μg/ml. The obtained PFD@NGHP was sufficiently reacted with GSH for 24 h, and the mixture was centrifuged at 4,000 rpm before detection. The UV absorption spectrum of PFD in water depicted a peak at about 308 nm and a shoulder peak at about 222 nm [38]. The absorbance at 308 nm of standard PFD solutions of the known concentration was obtained to make the calibration curve. Finally, the concentration of PFD released from different samples was calculated according to the standard calibration curve. The EE and EC were calculated by the following equations:

$$\mathrm{EE}=\frac{W_{\mathrm{PFD}\;\text{in NGHP}}\;}{W_{\mathrm{PFD}\;\text{feeded}}}\times 100\text{\%}$$ (1)

$$\mathrm{EC}=\frac{W_{\mathrm{PFD}\;\text{in NGHP}}\;}{W_{\text{NGHP}}}\times 100\text{\%}$$ (2)

The in vitro release of PFD from the NGHP in different conditions was performed in PBS with specified pH and reductive state. One milliliter of PFD@NGHP (1 mg/ml) was dialyzed with a membrane of 1-kD molecular weight cutoff in 50 ml of PBS. At the designed time point, 1 ml of release medium was withdrawn for detection and replaced with an equal volume of fresh medium. Drug quantitative analysis was performed as described above. Release profiles of PFD in 6 groups are reported for comparison. Results are expressed as release ratio over time ± SD of 3 experiments.

### HAase activity and content of NGHP

The enzyme assay and ELISA kit were used to determine the HAase activity and content of NGHP. As shown in our previous study [34], enzyme diluents (pH 7), hyaluronic solutions (pH 5.35), and acidic albumin solutions (pH 3.75) were prepared. Standard HAase solutions at different concentrations and NGHP solutions were swirled and incubated with 1 ml of hyaluronic solution at 37 °C in a water bath for 45 min. After 45 min, 0.5 ml of the above mixtures were transferred into 2.5 ml of acidic albumin solution and mixed immediately by inversion. The colloidal solution was formed after reaction, and its transmittance was determined at 600 nm to obtain a calibration curve. The HAase activity of NGHP can be calculated by the following equation:

$$\text{units}/\mathrm{ml}=\frac{\left(\text{\%}T\;\text{Test}-\text{\%}T\;\text{Blank}\right)\left(\mathrm{df}\right)}{14.84\left(\mathrm{ml}\;\text{of enzyme solution}\right)}$$ (3)

where df means the dilution factor of enzyme and 14.84 is the Sigma-Aldrich determined extinction coefficient.

The HAase content of NGHP was analyzed using a HAase ELISA kit (Mlbio, China). Samples were incubated in a 96-well plate for 1 h at 37 °C. After 3 washes, the substrate solution was added and incubated for 20 min at room temperature, followed by the addition of the stop solution. The optical density at 450 nm was measured using a microplate reader (BIOBASE, China).

### NGHP infiltration in ECM-mimicking gels

The infiltration ability of NGHP was assayed in ECM-mimicking gels. Building upon our established methodology [34], ECM-mimicking hydrogels were formulated with collagen I (9.37 mg/ml), HA (5 mg/ml in 2× PBS), and GSH (6.6 mM) as key constituents. The resultant gels contained final concentrations of collagen I (6.5 mg/ml), HA (1 mg/ml), and 200 μM GSH. After air bubble removal, 40 μl of gels were transferred into capillary tubes (R&D) and incubated overnight at 37 °C for gelation.

The FITC-labeled samples were used to reveal the infiltration trace in the experiment. FITC (DMSO solution) was added as a weight ratio of 10% into the NGP (nanogel–αPD-L1) and equivalent NGHP PBS solution (pH 8). Subsequently, the mixture was gently stirred for 8 h at 4 °C in the dark with the N_2_ protection. Finally, NGP–FITC and NGHP–FITC were obtained after dialysis using a membrane with a 1-kD cutoff molar mass in deionized water.

Twenty microliters of equalized NGP–FITC (1 mg/ml), NGP–FITC + free HAase (1 mg/ml), and NGHP–FITC solution (1 mg/ml) (the free HAase was equivalent to HAase of NGHP–FITC determined by the turbidimetric test) were slowly added to the capillary tube full of dense ECM-mimicking gels. The tube was sealed and incubated at 37 °C for 8 h in the dark. Then, tubes emitted the green fluorescence after exposure to the UV curing lamp (365 nm). The fluorescent trace was recorded by photo and then converted to numerical values using ImageJ. The nonlinear curve fitting was performed by OriginLab.

### Penetration ability evaluated by in vitro orthotopic tumor mass

To confirm the penetration ability of NGHP in tumor cultures, the orthotopic xenograft PAN02-enhanced green fluorescent protein (EGFP) tumor derived from the pancreas of C57BL/6 mice was surgically removed and passaged in Cultrex organoid-qualified BME, type 2. The tumor pieces were cultured in complete medium (advanced DMEM/F12 with 1% Hepes, 1% l-glutamine, and 1% penicillin and streptomycin) overnight. One day after tumor culture preparation, the tumor cultures of similar volumes were selected, and a layer of mimicked ECM composed of collagen I (5 mg/ml), hyaluronan (1 mg/ml), and 200 μM GSH was added. Subsequently, the culture systems were treated with Rho-labeled samples of different groups for 4 h. The NGP or NGHP was labeled by 6-carboxytetramethylrhodamine succinimidyl ester, as described in the previous work [34]. Two hundred microliters of equalized NGP-Rho (1 mg/ml), NGP-Rho + free HAase (1 mg/ml), and NGHP-Rho (1 mg/ml) (the free HAase was equivalent to HAase of NGHP-Rho, as determined by turbidimetric test) were used. After treatment, tumor cultures were washed twice and imaged with Z-stack model of CLSM.

### PD-L1 expression and avidity assay

CLSM and flow cytometry were used to evaluate the PD-L1 expression of pancreatic cancer cells. For CLSM, the live cancer cells and irradiated cancer cells were all stained to reveal the fluorescence of PD-L1. Cells in confocal dishes were incubated overnight at 37 °C, washed 3 times with cold PBS and blocked with 2% bovine serum albumin (BSA) in PBS. Subsequently, cells were incubated with avelumab (1 μg/ml) for 1 h at 4 °C. After incubation, cells were washed again and incubated with Alexa Fluor 488 goat anti-human IgG (H+L) cross-adsorbed secondary antibody (catalog no. A-11013) for 30 min in the dark at room temperature. Finally, cells were visualized by confocal microscopy after gentle washing. For flow cytometry method (FCM), cells were washed with PBS, blocked with 2% BSA solution, and incubated with avelumab (1 μg/ml) for 1 h at 4 °C. After removing the unbound antibody by washing with 2% BSA solution, cells were incubated with Alexa Fluor 488 goat anti-human IgG (H+L) cross-adsorbed secondary antibody for 30 min in the dark at room temperature, followed by washing with 2% FBS solution and analyzed by FCM. In addition, to compare with the avelumab, the PD-L1 binding avidity of NGHP was also assayed by FCM, as described above.

The avelumab ELISA kit of Abcam was used to quantify the antibody content of NGHP, and it can also reveal the affinity of NGHP. According to the protocol, the standard solutions and diluted samples were added to appropriate wells. After 0.5 h of incubation at room temperature, all wells were washed 3 times and tapped to remove residual liquid. Then, 100 μl of horseradish peroxidase (HRP) conjugate was added into each well, followed by incubation at room temperature for 30 min. After reaction, all wells were washed and tapped to be clear. Finally, the chromogenic reaction was developed with TMB substrate solution and stop solution, and the absorbance was read at 450 nm (reference wavelength to 650 nm).

### Detection of intracellular ROS

The intracellular ROS was measured via the 2′,7′-dichlorofluorescein diacetate (DCFH-DA)/Rosup double staining kit (Beyotime, China). We separately seeded hPSCs and mPSCs into a 24-well plate with DMEM/F12 containing 10% (v/v) FBS and 1% penicillin/streptomycin and incubated at 37 °C in a 5% CO_2_ atmosphere for 24 h. Subsequently, we replaced the culture medium and gave 6-Gy radiation to the designed groups. After 12 h, we washed the cells twice with PBS, added DCFH-DA solution, and incubated for 30 min. After incubation, we added Hoechst 33342 solution and incubated for 10 min. Finally, the production of ROS was observed through CLSM (Zeiss LSM 980), and background fluorescence from cell-free blank controls containing only DCFH-DA buffer was subtracted from all experimental groups.

### TGF-β1 detection via ELISA

The monoculture medium (PANC-1 cells, PAN02 cells, hPSCs, and mPSCs) and coculture medium (PANC-1&hPSC or PAN02&mPSC) were both assayed by TGF-β1 ELISA kit (Beyotime, China, #P878 and #P880). Moreover, the monoculture medium and coculture medium after 6-Gy radiation were also assayed. Before the detection, conventional culture medium or irradiated culture medium of different groups (control, NGHP, PFD, and PFD@NGHP) was collected and centrifuged at 1,000*g* for 10 min. Each sample was tested in 3 replicates. Following another wash, the substrate solution was added and incubated for 30 min at room temperature prior to the stop solution. A microplate reader (BIOBASE, China) was used to determine the optical density at 450 nm.

### Proliferation evaluated by CCK-8 assay

PANC-1 cells, hPSCs, PAN02 cells, mPSCs, and hTERT-HPNE cells were seeded, respectively, in 96-well plates 24 h before the assay. In addition, the PSC culture medium was pretreated with the addition of GSH to maintain a reductive environment. Due to obvious cytotoxicity of bPEI, the cells were treated with nanogel and NGHP for 48 h at different concentrations to determine the biocompatibility of nanocarriers. To determine the half-maximal inhibitory concentration (IC_50_) of PFD and PFD@NGHP, 1,000 to 2,000 cancer cells and PSCs in every well were treated with drugs at different concentrations. After 48 h of incubation, cell proliferation was analyzed with CCK-8 and read at an absorbance of 450 nm. IC_50_ values were calculated with OriginLab software. All measurements were performed in triplicate.

To analyze the effect of PFD after penetrating through the ECM, a Transwell assay was utilized to mimic the culture system in vitro. In the Transwell chamber, PSCs were seeded into the lower chamber and incubated overnight to achieve about 70% confluence. The upper chamber was coated with a mimicked ECM composed of collagen I, HA, and GSH and allowed for solidification in the cell incubator. Then, the upper chamber was plated on lower chamber, and samples were added into the upper chamber. After 4 h of sample diffusion through the ECM layer into the lower chamber, the upper chamber was removed, and the lower chamber was further incubated for 44 h. Finally, the CCK-8 assay was performed to analyze the proliferation of the remaining PSCs in the lower chamber.

To assess the inhibition of radiation combining with PFD or PFD@NGHP, cells were dosed with PFD (0.5 mg/ml) 6 h before radiation. During radiation, the 6-MV x-ray beam was coming from the vertical direction by a linear accelerator (Varian Medical System, USA) with a dose rate of 300 MU/min. Cells were irradiated with sham radiation (RT), 2, 5, 8, and 10 Gy, respectively. After radiation, cells were incubated for 48 h, followed by CCK-8 assay. The inhibition profile was assayed by OriginLab software.

### Organotypic 3D microtumors for radiotherapy and PFD@NGHP

To study the nanoparticle infiltration and subsequent colony inhibition due to the combinational treatment of PFD@NGHP and radiotherapy, the organotypic 3D microtumors derived from coculture system of PANC-1&hPSC or PAN02&mPSC were used to simulate the PDAC microenvironment. To generate the coculture 3D spheroids, the liquid overlay technique was used in the 96-well round-bottom ultralow adhesion (ULA) plates (Corning, Thermo Fisher Scientific, USA) that can promote cellular self-aggregation into a scaffold-free microtumor seeds. PANC-1 cells, hPSCs, PAN02 cells, and mPSCs were all detached with 0.25% trypsin/EDTA and resuspended in their respective culture medium. For the assembly of coculture PANC-1&hPSC spheroids, their cellular suspensions were prepared and seeded into 96-well round-bottom ULA plates with the density of 10,000 PANC-1 cells and 40,000 hPSCs per well. So, the ratio of PANC-1 cells to hPSCs was 1:4, and the cell mixture was gently pipetted to random distribution. Then, they were cultured under 5% CO_2_ atmosphere at 37 °C, and the culture medium was replaced every 2 d. Five days later, cells aggregated to form the microtumor seeds, and they were subsequently transferred to the basal Matrigel. As described previously, 400 μl of Matrigel (5 mg/ml) and hyaluronan (0.5 mg/ml) mixture was added to coat the bottom of the 24-well plate and then dried at 37 °C for 1 h to form basal Matrigel. Microtumor seeds were allowed to attach to the Matrigel overnight in an incubator. Finally, 400 μl of mimicked ECM composed of collagen type I (5 mg/ml), GSH (200 μM), and hyaluronan (1 mg/ml) in DMEM with 10% FBS was pipetted down to cover the basal Matrigel. This coculture 3D model was incubated at 37 °C overnight to form the stable system. On day 1, coculture 3D models were all treated with 6-Gy radiation. After that, the supernatant of the 3D model was removed before the dosing of 400 μl of PFD, PFD + HAase + αPD-L1, and PFD@NGHP solution at a concentration of PFD (1 mg/ml). HAase and αPD-L1 were also added in equal concentrations. After 4 h of treatment, the supernatant was removed to avoid sustaining infiltration of the samples, and 200 μl of DMEM with Matrigel (0.5 mg/ml) and 10% FBS was subsequently added into the plate cultures. The remained cultures with infiltrated samples were still incubated for 24 h. After concurrent therapy, all coculture 3D models continued to be cultured and were replaced with the surface Matrigel–DMEM every 2 d. On day 7, coculture 3D spheroids were checked and compared under microscopy. For PAN02 cell and mPSC cocultured 3D microtumors, the same procedure was implemented. The number and volume of spheroids in 100× field were calculated. Spheroids with a short diameter of >200 μm were be counted. Volume was estimated using the formula: Volume = Length × Width × Width / 2.

To assess the changes in cellular composition in the organotypic 3D microtumors, we distinguished the cellular components using EGFP-expressing cancer cells and red-fluorescent-labeled α-SMA of PSCs. At the beginning, the EGFP plasmid was transfected into PANC-1 and PAN02 cells. The EGFP-expressed PANC-1 cells and hPSCs were cocultured to generate 3D spheroids following the above protocol. After radiotherapy and PFD therapy, the coculture 3D models were rinsed gently and centrifuged to discard the supernatant on day 7. Then, the coculture 3D models were fixed with 4% paraformaldehyde, permeated with 0.2% Triton X-100, blocked with 5% BSA solution, and incubated with anti-α-SMA antibody (1:400) solution for 1 h at 4 °C. Finally, the coculture 3D models were incubated with Alexa Fluor 594 goat anti-rabbit IgG for 30 min, followed by immunofluorescent imaging. The fluorescence image of coculture 3D model containing PAN02-EGFP and mPSCs was also performed as described above. Finally, the ratio of red fluorescence area to green fluorescence area was calculated as the infiltration area ratio via ImageJ.

### Western blot analysis

PSC lysates were prepared using radioimmunoprecipitation assay lysis buffer containing protease and phosphatase inhibitors and kept on ice for 10 min. Protein concentration was determined using a bicinchoninic acid protein assay kit (Thermo Fisher Scientific, USA). About 30 μg of protein per sample was resolved by 10% SDS-polyacrylamide gel electrophoresis and electrotransferred to 0.45-μm polyvinylidene difluoride membranes of Millipore. These membranes were then blocked with 5% BSA solution at room temperature for an hour, cut for probing various proteins, and incubated with primary antibodies overnight at 4 °C. The primary antibodies utilized are specified as described above. Following thorough washing steps and incubation with secondary antibodies, detection was performed via an ECL system.

### Antibody-dependent cell-mediated cytotoxicity assay

The CytoTox 96 nonradioactive cytotoxicity assay was carried out to evaluate the antibody-dependent cell-mediated cytotoxicity (ADCC) induced by avelumab. The human peripheral blood mononuclear cells (PBMCs) were purchased from iXCells Biotechnologies (CA, USA). PBMC effectors were allowed to rest overnight in RPMI 1640 medium containing 10% heat-inactivated FBS (Gibco). PANC-1 cells and irradiated PANC-1 cells were used as targets, respectively, at a working concentration of 20,000 cells per well in 96-well round-bottom ULA plates. PBMCs were added to the cancer cell wells at effector-to-target cell ratios of 10:1. The working concentration of simple avelumab and avelumab of NGPH was 10 μg/ml, with the similar equivalent amount of HAase detected in the specific group. Given the obvious cytotoxicity of the naked nanogels, mPEGS-modified nanogels were used to evaluate. The mixed cells were incubated for 4 h at 37 °C with 5% CO_2_ and centrifuged at 250*g* for 5 min after incubation. Fifty microliters of supernatant was removed from each well and transferred to 96-well plate to react with 50 μl of working solution for 30 min in the dark. Finally, the absorbance of each well was read at 490 nm following reaction stop. Each sample was tested in 3 replicates. Cytotoxicity was determined using:

$$\text{Cytotoxicity}=\frac{\text{Experimental}-\text{Effector Spontaneous}-\text{Target Spontaneous}}{\text{Target Maximum}-\text{Target Spontaneous}}$$ (4)

### In vivo tumor inhibition study and abscopal effect assessment

All experimental animals were maintained under specific-pathogen-free conditions. To analyze the antitumor efficacy in vivo, PAN02 cells (1 × 10^6^) and mPSCs (1 × 10^6^) were mixed and subcutaneously transplanted into the right flank of 5-week-old male C57BL/6 mice. About a week after implantation, mice were randomly divided into 8 groups (5 mice per group): control, PFD, PFD + HAase + αPD-L1, PFD@NGHP, RT, RT + PFD, RT + PFD + HAase + αPD-L1, and RT + PFD@NGHP. The PBS, PFD@NGHP, equivalent amount of PFD (10 mg/kg), HAase (6.5 mg/kg), and αPD-L1(avelumab; 12 mg/kg) were all used via intravenous injection from day 1 to day 9, every other day (qod). For radiotherapy, a 6-MV x-ray photon beam was delivered to the exposed tumor of the securely immobilized mice using a specially designed model. The target field of 40 cm × 4 cm only covered the tumors and avoided the organs at risk. Mice were treated with 6 Gy on day 1 and day 3. Then, tumor size and mouse body weight were monitored and recorded every 3 d throughout the whole experiment. Finally, mice were euthanized, and tumors were removed from the mice to be weighed. The tumors were further excised and subjected to further analysis including flow cytometry analysis, hematoxylin and eosin (HE), immunohistochemistry, and immunofluorescence. The major organs were fixed and then stained with HE to evaluate the overall biosafety of the nanoparticles.

To monitor the abscopal effect in vivo, the primary tumor of PAN02 cells/mPSCs was established in the right flank, as described above. When the primary tumor volume reached about 150 mm^3^, PAN02 cells were inoculated into the left flank to establish the distal tumors. About 5 d later, mice were randomly divided into 4 groups: RT, RT + PFD, RT + PFD + HAase + αPD-L1, and RT + PFD@NGHP. As described above, the PBS, PFD@NGHP, PFD, HAase, and αPD-L1 were all injected from day 1 to day 9, qod. Focal radiotherapy (6 Gy) was delivered to the exposed tumors on the right flank on day 1 and day 3, while the distal tumors were shielded with lead. Then, tumors of both flanks were monitored and recorded every 3 d throughout the whole experiment.

### Immunohistochemistry and immunofluorescence assay of tumor

For immunohistochemical staining, paraffin-embedded tissue slides were deparaffinized with xylene for 30 min and rehydrated through a graded ethanol series (100%, 90%, 80%, 70%, and 50%) and water for 5 min each. The slides were placed in sodium citrate buffer for antigen retrieval for 20 min (with microwave heating). After blocking with 3% BSA, the slides were incubated with a primary antibody overnight at 4 °C in a humidified chamber. The slides were then incubated in a 37 °C water bath for 30 min and washed 3 times with PBS. Subsequently, HRP-conjugated secondary antibodies incubation was conducted at room temperature for 1 h. After counterstaining with hematoxylin and developing with 3,3′-diaminobenzidine, the slides were observed under a microscope (Nikon, Japan).

For immunofluorescence staining, slides were prepared to be blocked for 30 min, as described above. Then, the slides were incubated with the first primary antibody at 4 °C overnight. After incubation, the slides were washed 3 times by PBS on a destaining shaker for 5 min each time. The slides were dried, and the HRP-labeled secondary antibody conjugated with the corresponding species of the primary antibody was added and incubated at room temperature for 50 min. After washing and drying, CY3-TSA was added and incubated at room temperature in the dark for 10 min. Before the second immunofluorescence staining, the slides were subjected to antigen retrieval with microwave heating again. Then, the above staining procedures were repeated to stain the FITC, CY5, or/and DAPI. Finally, the slides were mounted with an antifluorescence-quenching mountant and scanned by confocal microscopy (Carl Zeiss) (DAPI, blue light; FITC, green light; CY3, red light; CY5, red light).

### In vivo evaluation of T cell infiltration

Tumors from different groups were enzymatically digested with collagenase IV, deoxyribonuclease I, and HAase to generate a single-cell suspension. The resulting cell suspension was filtered through a 70-μm strainer and washed with FCM buffer. Subsequently, 1 million cells were stained with Fixable Viability Stain 780 in FCM buffer for 30 min at 4 °C. The cells were then washed twice with FCM buffer to remove excess dye and incubated with anti-CD3-FITC, anti-CD8-PE, and anti-CD4-APC antibodies for 30 min at room temperature in the dark. Finally, the cells were washed twice and resuspended in FCM buffer for flow cytometry analysis.

### Statistical analysis

Statistical analyses were performed using R version 4.2.1, using either Student’s *t* test or one-way analysis of variance (ANOVA) to determine statistically significant differences. Differences were considered significant at a *P* < 0.05, whereas *P* > 0.05 represents nonsignificant differences.

## Results and Discussion

### Hypersecretion of TGF-β1 and overexpression of PD-L1 induced by radiation

**Fig. 2.**
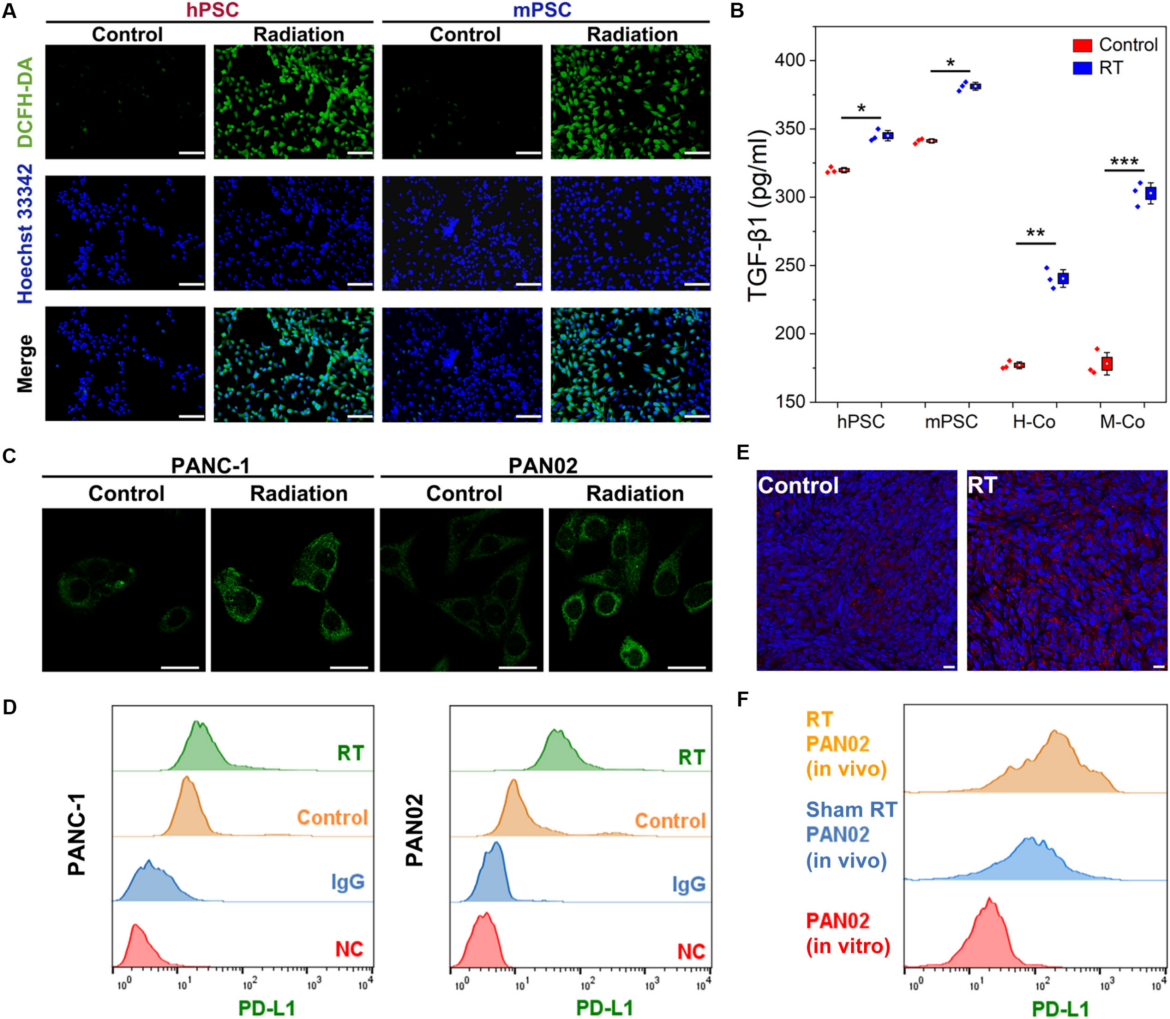
TGF-β1 secretion and PD-L1 expression induced by radiotherapy at cellular and tissue level. (A) Immunofluorescent images produced by ROS in PANC-1 and PAN02 cells treated with or without irradiation. Scale bars, 100 μm. The blue fluorescence shows the cell nucleus, and the green fluorescence represents ROS. (B) TGF-β1 secretion from monoculture PSCs (both human and mouse) and coculture systems (PANC-1&hPSC and PAN02&mPSC) under radiotherapy and nonradiotherapy conditions was assessed by ELISA. (C) CLSM images and (D) FCM analysis of PD-L1 expression on PANC-1 and PAN02 cells under radiotherapy and nonradiotherapy conditions. The green fluorescence represents the expression of PD-L1 on cancer cells in CLSM images. Scale bars, 20 μm. (E) The PD-L1 expression in PAN02 mouse xenograft tumors was detected by immunofluorescence assay, with PD-L1 expression displayed as red fluorescence. Radiation induced the up-regulation of PD-L1 expression in PAN02 tumors. Scale bars, 20 μm. (F) The PD-L1 expression of PAN02 (in vitro), PAN02 (in vivo), and irradiated PAN02 (in vivo) was compared using FCM. The result indicated the increased expression in irradiated PAN02 cells in vivo.

Ubiquitously, ionizing radiation interacts with water molecules to generate ROS that is a critical mechanism underlying radiation-induced cell damage. As shown in Fig. 2A, Hoechst 33342 marked cell nuclei to identify viable hPSCs and mPSCs. Normal PSCs presented sparse green fluorescence of DCFH-DA detecting ROS, while the irradiated PSCs all presented intense green fluorescence reflecting the ROS accumulation. In addition, this ROS accumulation is strongly correlated with the observed elevation in TGF-β1 secretion, as depicted in Fig. 2B. Quantitative measurements by ELISA revealed significantly higher levels of TGF-β1 in irradiated PSCs (hPSCs and mPSCs) and coculture systems (H-Co and M-Co) compared with the levels of TGF-β1 in the control group. TGF-β1 was almost undetectable in pancreatic cancer cells (PANC-1 and PAN02), even in irradiated cancer cells (Fig. S1), which had minimal impact on the detection results in the coculture systems. As explained by numerous studies, the generation of ROS triggered the release of TGF-β1 through the activation of various enzymatic pathways, such as NOXs, COX-2, and iNOS [24]. The radiation-induced accumulation of ROS in PSCs significantly elevated TGF-β1 release, potentially contributing to tumor progression or stromal fibrosis. This could consequently impede the efficacy of radiotherapy.

In addition to its effects on PSCs, RT promoted PD-L1 expression in pancreatic cancer cells (PANC-1 and PAN02). In the in vitro experiment, strong PD-L1 fluorescence intensity was observed in irradiated cancer cells (Fig. 2C), which was further corroborated by the flow cytometry data shown in Fig. 2D. The flow cytometry revealed a noticeable rightward shift in the PD-L1 expression distribution within the radiation group, particularly evident in PAN02 cells. Moreover, it extended these observations in vivo in Fig. 2E and Fig. S2. Xenografted tumor tissues of PAN02 cells, derived from C57BL/6 mice, exhibited significantly higher PD-L1 expression after radiotherapy, as evidenced by semiquantitative analysis in Fig. S2. Finally, the comparative flow cytometry analysis also revealed the up-regulated PD-L1 expression in PAN02 cells in vivo. Furthermore, the PD-L1 expression was significantly elevated in the postradiotherapy tumors (Fig. 2F and Fig. S3). These results aligned with the classic mechanism of PD-L1 up-regulation in tumor cells following radiotherapy. DNA damage activated signaling pathways such as ATM-ATR/Chk1 and the cGAS/STING pathway, leading to increased PD-L1 expression in in vitro tumor cells. However, the immune response triggered by damage-associated molecular patterns further enhanced PD-L1 expression in vivo, reinforcing an immunosuppressive TME. This sequential activation of both intrinsic DNA damage pathways and extrinsic immune responses explains the higher levels of PD-L1 expression observed in tumors after radiotherapy. Therefore, these stromal and immunological changes in TME induced by irradiation would complicate the efficacy of radiotherapy, and colocalized and simultaneous modulation of these adverse changes may potentially improve therapeutic outcomes.

### Synthesis, characterization, and biocompatibility of PFD@NGHP

**Fig. 3.**
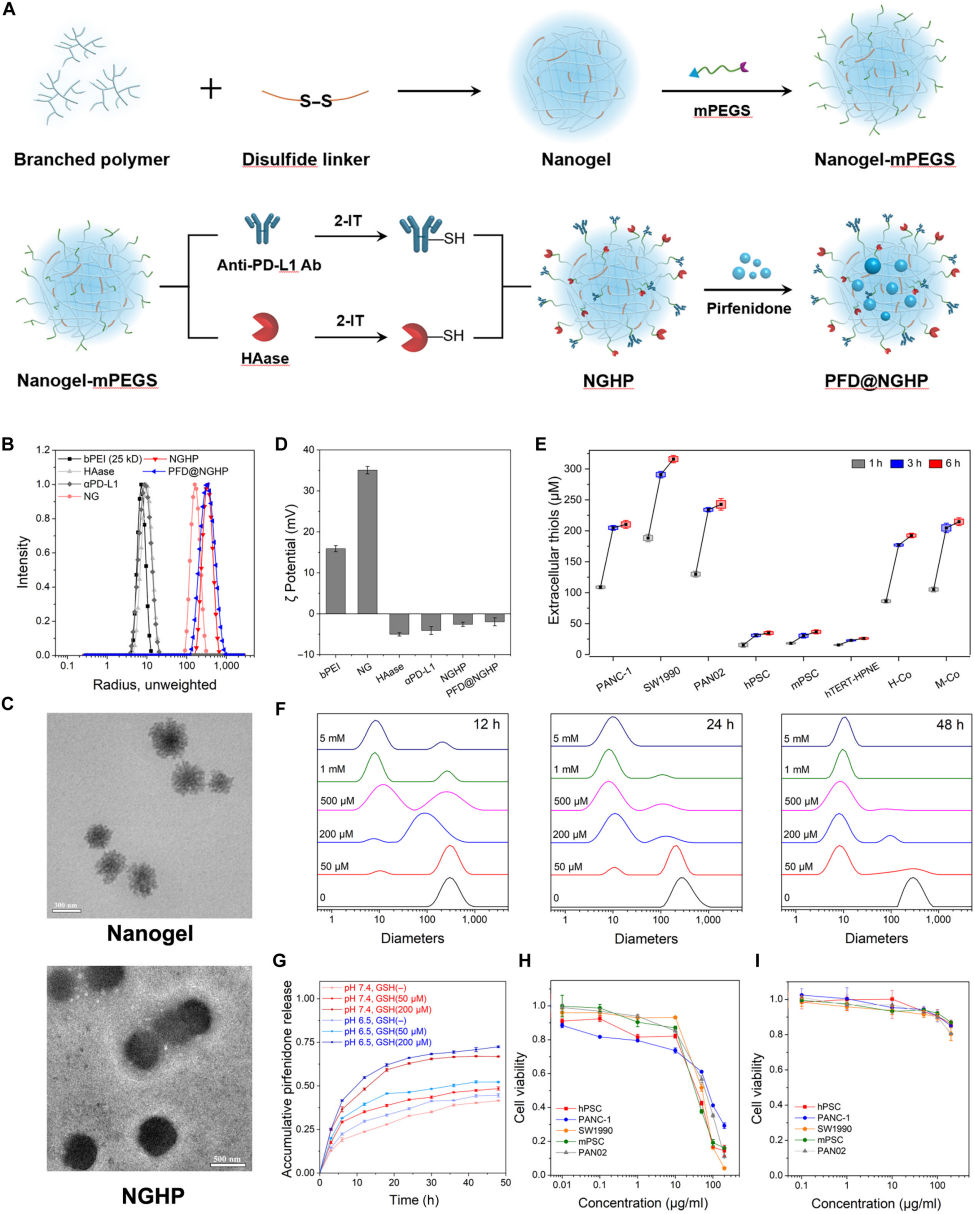
The physicochemical characteristics, release profile, and biocompatibility of nanogel system. (A) Synthesis scheme of PFD@NGHP. Ab, antibody. (B) DLS analysis of bPEI, HAase, αPD-L1, nanogel (NG), NGHP, and PFD@NGHP. The hydrodynamic radii of bPEI, HAase, αPD-L1, nanogel, NGHP, and PFD@NGHP were 5.8, 7.9, 7.2, 161.2, 332.1, and 318.6 nm, respectively. (C) Nanogel and NGHP were imaged by transmission electron microscopy. Scale bars, 300 nm. (D) Zeta potential values of bPEI, HAase, αPD-L1, nanogel, NGHP, and PFD@NGHP. Values indicate means ± SD (*n* = 3). (E) Extracellular thiols from different cell culture systems assayed at 1, 3, and 6 h after the addition of fresh medium. (F) DLS analysis of size distributions at different GSH concentrations and reaction times revealed the redox sensitivity of the nanogels. (G) The release profiles of PFD@NGHP in different reductive and pH environments were evaluated. Various GSH concentrations and pH levels were used to mimic the extracellular reductive environments of tumors or normal tissues. (H) Nanogels and (I) NGHP exhibited different biocompatibilities in pancreatic cancer cells and PSCs. The concentration of bPEI was set as the reference standard.

With reference to our previous work [34], bPEI chains were linked by DSP to formulate PEI nanogels. According to the TNBS assay, the residual primary amines exposed on PEI nanogels after cross-linking were determined (Fig. S4A and B). It was calculated that the exposed primary amines of PEI nanogels accounted for 45.6% of the initial input concentration of primary amines from bPEI. The depletion of primary amines resulted from several factors, including DSP consumption, removal of small PEI nanogels during dialysis and potential steric shielding effects. However, the remaining primary amines are adequate for further use. mPEGS was then used to anchor the sulfhydryl HAase and sulfhydryl antibodies on nanogels to obtain NGHP. Finally, PFD was added to the NGHP solution and absorbed into nanogels to obtain PFD@NGHP (Fig. 3A).

As we know, the physical and chemical properties of nanogels, such as *R_g_*, *R_h_*, *Mw*, *A*_2_, and *ρ*, strongly influence their in vivo application. The structure of PFD@NGHP was carefully tailored, and its physicochemical properties were thoroughly characterized using LLS. The size distributions of NGHP and its compositions were analyzed by DLS and are summarized in Fig. 3B. The hydrodynamic radii (*R_h_*) of bPEI (25 kD), HAase, αPD-L1, nanogel, NGHP, and PFD@NGHP were 5.8, 7.9, 7.2, 161.2, 332.1, and 318.6 nm, respectively. Obviously, the *R_h_* increased from nanogel to NGHP due to the conjugation with HAase and αPD-L1. NGHP maintained a similar size (*R_h_* = 312.2 nm) after incubation in conditional DMEM of 10% FBS for about 48 h, which, to some degree, determined the superior stability of NGHP under serum-mimicking conditions (Fig. S5A). After that, the particle size distribution of NGHP became broader and gradually merged with the peak of FBS, possibly indicating the gradual degradation of NGHP. SLS was used to analyze the properties of *Mw*, *A*_2_, and *ρ*(*R_g_*/*R_h_*). To satisfactorily fit the SLS data and reduce errors of extrapolation, we applied the Berry method for calculating the *Mw*, *A*_2_, and *ρ* of the NGHP (Fig. S5B). The *R_g_* of the NGHP is 298.8 nm. The *Mw*, *A*_2_, and *ρ* of the NGHP were 1.678 × 10^7^, 5.256 × 10^−9^, and 0.899, respectively. According to the corresponding ELISA assay, HAase was determined to be 112.3 ± 0.2 μg/ml, and the αPD-L1 was determined to be 235.1 ± 0.4 μg/ml in NGHP solution (0.5 mg/ml). Due to the large amounts of HAase and αPD-L1 conjugated to the surface of the core nanogel via mPEGS, the *ρ* reached 0.899, indicating that the NGHP exhibited a spherical shape with inhomogeneous density.

It is consistent with the core–shell morphology observed in TEM image. The TEM image of nanogels exhibited a spherical morphology with a branched structure, measuring approximately 300 nm in diameter (Fig. 3C). The NGHP exhibited a dense spherical core with branching structures and a distinct halo-like surface, likely representing the HAase and αPD-L1 shell. Its size of about 600 nm also closely matched the observations in TEM. Surface potentials of the NGHP and its components were summarized in Fig. 3D. The original nanogel presented a strong positive charge (35.1 ± 0.9 mV), while the HAase and αPD-L1, being negatively charged biomacromolecules, reduced the overall zeta potential upon linking to the nanogel (−2.6 ± 0.5 mV). This indicates successful assembly and the electrostatic interaction between components, which confirmed the structural integrity of the PFD@NGHP. Importantly, the surface potential of NGHP was effectively reduced after modification with HAase and αPD-L1, facilitating appropriate in vivo circulation.

High redox level in tumor mass and intracellular system can be exploited for drug delivery in cancer [39]. However, in recent years, some further evidence has been obtained that certain reductive processes can also take place in extracellular regions due to the presence of extracellular thiols from cysteine or GSH [39,40]. More extracellular thiols or GSH was confirmed in the culture medium of cancer cells, including prostate cancer (RWPE1 and WPE1-NB26), lung cancer (A549), liver cancer (HepG2), etc. [41,42]. Similarly, more extracellular thiols were also detected in culture medium of pancreatic cancer cells in our study. The extracellular thiol concentrations over time in different cell types were presented in Fig. 3E. Tumor cells (PANC-1, SW1990, and PAN02) exhibited higher thiol levels compared to normal or stromal cells, such as hPSCs, mPSCs, and hTERT-HPNE cells. Within hours of culture, the concentration of extracellular thiols in the culture medium from tumor cells or coculture system reached nearly 200 μM, whereas in PSCs and hTERT-HPNE cells, the extracellular thiol levels remained below 50 μM. This high extracellular reductivity in the TME is essential for the selective disassembly of nanogel.

To evaluate the reductive responsiveness of nanogels, we set up the various reductive environments using GSH. As shown in Fig. 3F, size distributions at different GSH concentrations and at different time points were classified and compared. The size distribution shifted to smaller diameters with increasing GSH concentrations, reflecting the progressive cleavage of disulfide bonds and disintegration of the nanogel. The disassembly led to the release of smaller fragments or PEI monomers, which accounted for the 2 distinct size distributions observed under certain conditions. High GSH levels, such as 1 and 5 mM, quickly induced the reduction of disulfide bonds, leading to the disruption of nanogels within 12 h, mimicking the conditions typically found in the intracellular environment. Notably, at a GSH concentration of 200 μM (mimicking tumor extracellular conditions), the nanogel completely dissociated within 24 h, whereas at 50 μM (mimicking normal tissues), the nanogel remained intact for a longer period. Therefore, extracellularly secreted thiols, reaching concentrations of about 200 μM, were capable of disassembling the nanogels within an appropriate time frame. In addition, this result was consistent with the data reported by some studies [39,43].

Importantly, the release profile of PFD@NGHP was also studied to evaluate its drug delivery effect. The PFD encapsulation of NGHP was first determined by UV spectrophotometer. As shown in Fig. S6A, the UV absorption spectrum of PFD in water depicted *λ*_max_ at about 308 nm and a shoulder peak at 222 nm. The calibration curve in Fig. S6B was fitted from the scan data of PFD standard solutions. The UV absorption spectra of PFD, NGHP, and PFD@NGHP were compared in Fig. S6C, and the peak of PFD could be distinguished from the peak at 280 nm of proteins. The calculated value of EE was 9.81 ± 0.14%, and the EC was 39.02 ± 0.35%. The release profile of PFD@NGHP was then studied under different environmental conditions, specifically varying pH and GSH concentrations, which roughly mimicked TMEs and normal tissue conditions. As shown in Fig. 3G, in a normal pH of 7.4 with no GSH, the release profile of PFD was relatively slow, indicating limited drug release. As the GSH concentration increased to 50 μM, there was a slight acceleration in the release rate (about 30% PFD in 10 h). Interestingly, a significant acceleration was observed when the GSH concentration increased to 200 μM (dark-red line, about 40% PFD in 10 h), revealing a pronounced promotion compared to the concentrations of 0 and 50 μM. Furthermore, at a lower pH of 6.5 (simulating the acidic environment in tumor), the PFD release was faster, even without GSH. The release profile of PFD was the fastest (about 50% PFD in 10 h) at pH 6.5 when the GSH concentration increased to 200 μM, suggesting that the PFD@NGHP formulation is sensitive to the TME with lower pH and higher GSH concentrations. In acidic reductive environments, disulfide bonds are more prone to cleavage due to 2 main factors [44]. First, hydrogen ions under acidic conditions protonated the sulfur atom, weakening the bond. Second, in reductive environments, GSH donated electrons to reduce the sulfur atoms and break the bond. As a result, the PEI framework tended to break down in these environments, facilitating the easy diffusion of PFD from the core to the exterior [44]. Hence, these results indicated that PFD@NGHP could be efficient in PFD release in the TME.

The biocompatibility of nanogel and NGHP was evaluated in hPSCs, PANC-1 cells, SW1990 cells, mPSCs, PAN02 cells, and hTERT-HPNE cells. The nanogels exhibited dose-dependent cytotoxicity due to the cationic nature of bPEI, which can disrupt cell membranes and induce obvious cell death at concentrations above 10 μg/ml (Fig. 3H and Fig. S7). Although different cell types exhibited varying degrees of tolerance to PEI toxicity, most cells shown about 50% viability around a concentration of 50 μg/ml. However, the cytotoxicity is significantly reduced when the nanogels are conjugated with antibodies and HAase. This coating reduced the surface charge of the nanogels, which prevented direct interaction of the core–nanogel with the cell membrane, thereby reducing toxicity. As shown in Fig. 3I, even the core–nanogel of NGHP reaching the concentrations of 100 μg/ml, cells were all metabolically active (about 90% survival rates). These assays highlighted the enhanced biocompatibility of the nanogels when surface-modified, indicating that the formulated NGHP improved the safety profile for biomedical applications at the appropriate concentration. According to this result, the NGHP will not mask the cell inhibition effects of PFD.

### The penetration ability and of proliferation inhibition of PFD@NGHP

**Fig. 4.**
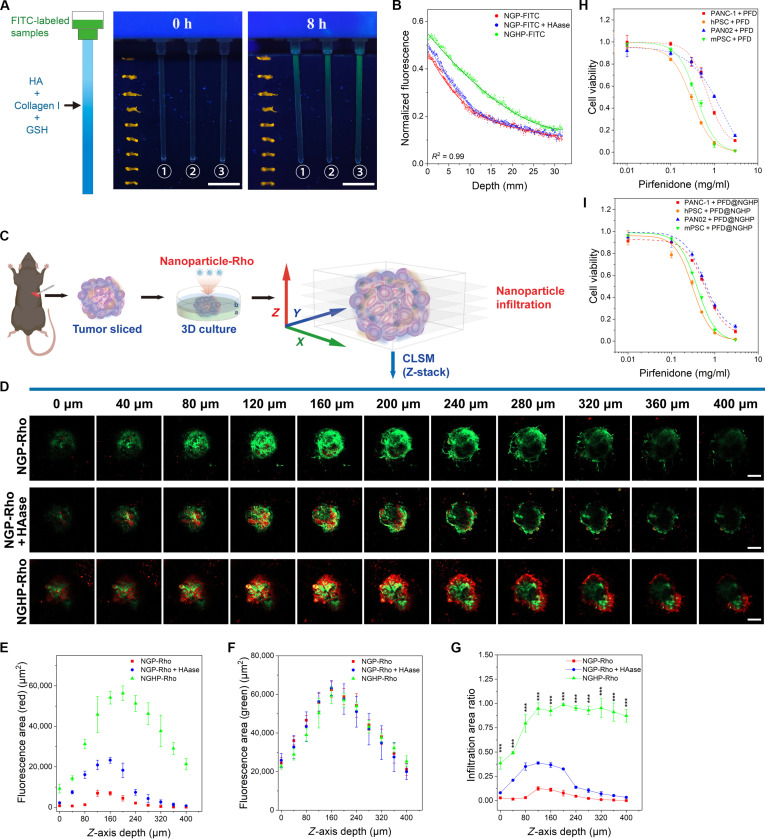
The penetration ability assay of nanogel systems via biomimetic 3D models and proliferation inhibition profile of PFD@NGHP. (A) Penetration ability of FITC-labeled nanogel systems assayed in mimicked ECM of capillary tube. The green fluorescence in capillary tube was excited by UV light after 8 h of incubation and imaged to quantitative fluorescence distribution. Scale bars, 1 cm. (B) Normalized FITC fluorescence of different nanogel systems, converted from fluorescent image in (A), was fitted by nonlinear curve model in OriginLab. *R*^2^ was 0.99. (C) Establishment and application schematic of orthotopic tumor in vitro culture model. (D) The penetration ability of Rho-labeled nanogel systems assayed in orthotopic tumor in vitro culture model. The in vitro cultured orthotopic tumors were imaged slice by slice via Z-stack model in CLSM to evaluate the distribution of Rho-labeled nanogel systems. Scale bars, 200 μm. (E) Normalized red fluorescence area, representing tumor infiltration of different nanogel systems, was semiquantified from fluorescent image in Fig. 3D. Data are presented as means ± SD. (F) Normalized green fluorescence area, representing tumor tissues of different groups, was semiquantified from fluorescent image in Fig. 3D. Data are presented as means ± SD. (G) The ratio of red fluorescence area to green fluorescence area was calculated as the infiltration area ratio, which approximates the infiltration degree of different nanogel systems at specific depths. Data are presented as means ± SD; ***P < 0.001 as compared to group of NGP-Rho + HAase. (H) PFD and (I) PFD@NGHP exhibited different proliferation inhibitions on PANC-1 cells, hPSCs, PAN02 cells, and mPSCs. The IC_50_ values of PFD and PFD@NGHP in different cell lines were calculated.

The potent permeability of PFD@NGHP in stroma was derived from its surface-conjugated HAase. The HAase activity of NGHP was first determined according to the calibration curve in Fig. S8 obtained from the turbidimetric test. The enzyme activity was calculated as 243.7 ± 5.2 units per 1 mg of NGHP, which could generate ablation of HA during the permeation process. To quantify the penetration effect of NGHP in stroma, we deployed gels composed of collagen I, HA, and GSH to simulate tumor ECM. As shown in Fig. 4A, the fluorescence of the entire capillary tube in all 3 groups was individually detected and recorded after 8-h incubation at 37 °C. The experimental setup included 3 groups: NGP–FITC, NGP–FITC + HAase, and NGHP–FITC, while the corresponding yellow markers indicated the depth within the capillary. In the group of NGP–FITC (“①” in Fig. 4A), the green fluorescence was primarily confined to the upper half of the capillary, with a penetration depth of about 1 cm. As the depth increased, the fluorescence intensity decreased rapidly, indicating limited penetration in the capillary. In the middle group of NGP–FITC + HAase (“②” in Fig. 4A), the fluorescence intensity, to some degrees, was visibly higher than that of the NGP–FITC group. However, the fluorescence was still concentrated around 1 cm in depth, and beyond that point, the fluorescence dimmed and became almost invisible. In the right group of NGHP–FITC (“③” in Fig. 4A), a significantly higher penetration depth of up to 2.5 cm was observed, with visible green fluorescence infiltrating much deeper into the capillary compared to the other 2 groups. To provide a more objective analysis of the fluorescence distribution along the capillary, we calibrated the fluorescence intensity values for each group based on the initial fluorescence intensity of the samples by OriginLab. After nonlinear fitting of the data, the penetration curves for each group provided a clearer representation of nanogel permeation (Fig. 4B). As shown in Fig. 4B, the normalized fluorescence intensity of NGHP–FITC seemed to be relatively higher compared to the other 2 groups, and the curve exhibited a flatter trend. The visible penetration distance in the NGHP–FITC group was about twice that of the other 2 groups, which reflected superior penetration capacity of NGHP. In contrast, the NGP–FITC + HAase group exhibited the higher normalized fluorescence intensity than that of the NGP–FITC group within the first 1 cm, but the fluorescence intensity decreased rapidly within this range, following a similar trend to the NGP–FITC group. In addition, the fluorescence intensity below 1 cm primarily originated from background light, suggesting the limited penetration profile of nanogels in this group. In summary, the NGHP–FITC exhibited the most effective permeation, with a deeper and more consistent fluorescence distribution across the capillary. These results suggested that the simple mixture of NGP–FITC and HAase had only a slight impact on the deeper diffusion due to the spatial and temporal inconsistency leading to a suboptimal penetration. However, NGHP–FITC, conjugating the HAase with NGP, ensured consistency between NGP and HAase during the penetration process, which facilitated superior penetration efficacy in the ECM-mimicking gels and probably in the stroma.

To determine the stromal penetrability of NGHP, we cultured the orthotopic tumors of PAN02-EGFP cells and served them as the validation model (Fig. 4C). As shown in Fig. 4C, tumor cultures were incubated in layer “a” and coated with layer “b”. Then, the culture system was incubated with Rho-labeled nanoparticles and scanned section by section via CLSM. The scanned slices from 0 to 400 μm of 3 groups were all presented in Fig. 4D. In addition, the semiquantitative analysis based on the different fluorescence in Fig. 4D was presented in Fig. 4E to G. The green fluorescence of the tumor tissues changed from dark to bright and then back to dark as the depth increased, which was correlated with the spatial changes in green fluorescence area depicted in Fig. 4F. Due to the 3D culture, the fluorescence at the center of the solid tumor was relatively weak, while the fluorescence in the outer surface of the tumor was comparatively strong. Blocked by ECM barrier, NGP-Rho exhibited relatively poor infiltration into culture system, with the red fluorescence localized mainly in superficial sections (Fig. 4D). In addition, the deeper tumor sections lacked any bright-red fluorescence. According to the Fig. 4E, the peak penetration coverage of NGP-Rho was probably at the depth of 120 μm in 3D microtumors. The NGP-Rho and HAase combined group revealed a more extensive infiltration compared to NGP-Rho alone. HAase facilitated a moderate penetration of NGP-Rho into deeper tumor tissues. In the superficial sections, the red fluorescence was bright and widespread. Partial overlap of red fluorescence with the green fluorescence of PAN02 cells resulted in the appearance of yellow regions, suggesting some degree of colocalization. Although the maximum depth of infiltration in this group reached at about 240 μm, this infiltration remained somewhat heterogeneous. In addition, its peak penetration coverage may be at the depth of about 160 μm. Interestingly, a much more widespread red fluorescence of NGHP-Rho was observed in the superficial sections of microtumor. As the depth increased, the infiltration zone expanded without the decrease in fluorescence intensity. Even in deeper sections, large areas of yellow and red fluorescence were visible, corresponding to effective colocalization of NGHP-Rho with tumor cells. Although red fluorescence in the tumor center was very weak in the last few sections, it was still detectable in the tumor surface, with broad infiltration observed in some regions. The depth of infiltration reached beyond 400 μm, and its peak penetration coverage was at the depth of about 200 μm (Fig. 4E), which confirmed that NGHP-Rho possessed the highest penetration capacity.

Considering the instability caused by the spatial heterogeneity of the tumor stroma, we added mimicked ECM to filter the corresponding nanogels. Since the nanogels infiltrated from top to bottom, the nanogels reaching the tumor surface would exhibit distribution differences due to different permeation abilities. As reflected by the infiltration area ratio calculated from the red/green fluorescence ratio (Fig. 4G), the NGHP-Rho group sustained a stable, elevated plateau in infiltration area ratio beyond 120 μm, contrasting with the rapid decline observed in the other 2 groups. All the results demonstrated the strong permeation ability of NGHP both in the mimicked ECM and in the tumor stroma. Based on these findings, NGHP may be a promising candidate for improving the drug delivery and therapeutic efficacy of nanomedicines in solid tumors.

To ensure that the developed PFD@NGHP applied for cancer therapy, we evaluated the intrinsic cytotoxicity of PFD or PFD@NGHP in PANC-1 cells, hPSCs, PAN02 cells, and mPSCs. As shown in Fig. 4H and I, cells were differentially sensitive to PFD and PFD@NGHP. In this test, the toxicity possessed by NGHP would be ignored according to the above result, which could not affect the therapeutic effect of PFD. The proliferation inhibition was positively correlated with the concentrations of PFD and PFD@NGHP. After fitting and calculating, the IC_50_ values of PFD for different cells were IC_50_(PANC-1) = 0.696 mg/ml, IC_50_(hPSC) = 0.304 mg/ml, IC_50_(PAN02) = 1.018 mg/ml, and IC_50_(mPSC) = 0.396 mg/ml. For PFD@NGHP, IC_50_(PANC-1) = 0.597 mg/ml, IC_50_(hPSC) = 0.312 mg/ml, IC_50_(PAN02) = 0.572 mg/ml, and IC_50_(mPSC) = 0.393 mg/ml. Since PFD is an effective antifibrotic drug that is commonly used in the treatment of idiopathic pulmonary fibrosis, it would exert a stronger inhibitory effect on stellate cells compared to tumor cells [29]. Then, a final concentration of PFD (0.3 mg/ml) was applied in the subsequent experiments, which might exert a moderate inhibitory effect on tumor cells. PFD@NGHP exhibits potent stroma-penetrating capabilities coupled with marked PSC inhibition, potentially inducing a comprehensive stromal amelioration through an “inside-out” mechanism.

### The multifunctional biological therapeutic effect of PFD@NGHP

**Fig. 5.**
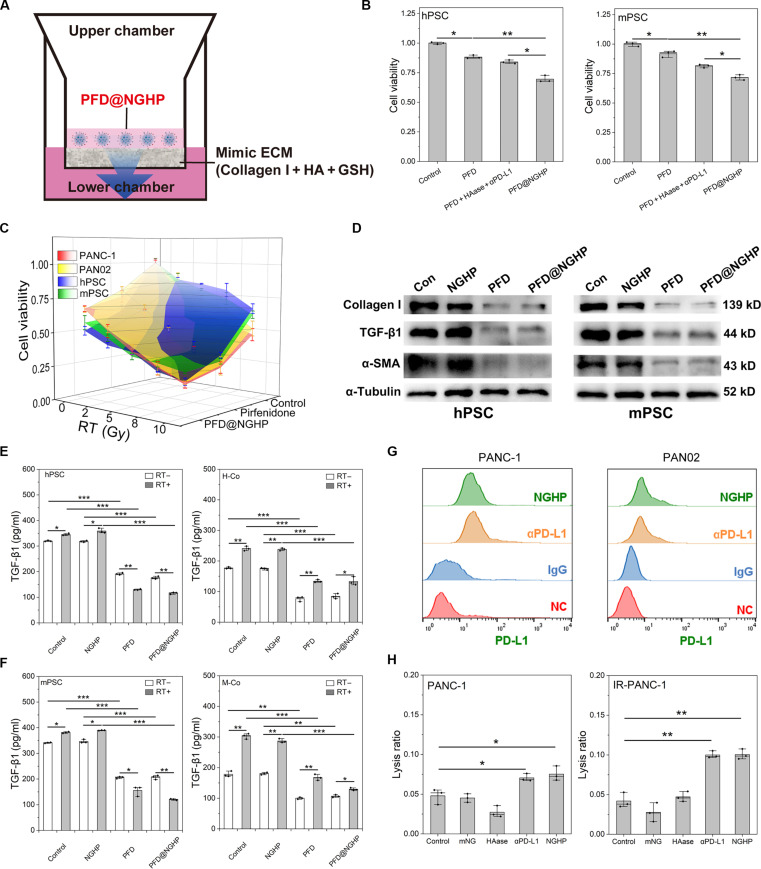
The biological multifunctional therapeutic effect of PFD@NGHP was assessed through a comprehensive evaluation. (A) Establishment schematic of Transwell assay system to evaluate the proliferation inhibition of PFD@NGHP. (B) Cell viability of PSCs determined by Transwell assay system. PFD@NGHP exhibited the most potent inhibitory profile. (C) Cell viability of different cell lines (PANC-1 cells, hPSCs, PANC02 cells, and mPSCs) was assessed via CCK-8 kit after different treatments including combination therapy or monotherapy. (D) Expression levels of collagen I, TGF-β1, α-SMA and α-tubulin in hPSC and mPSC lines treated under different conditions for 48 h, as determined by Western blotting. Con, control. (E) TGF-β1 secretion of hPSC and human cell coculture system (H-Co) under different conditions was evaluated by ELISA. Irradiated (RT+) cells and unirradiated (RT−) cells were both analyzed. (F) TGF-β1 secretion of mPSC and mouse cell coculture system (M-Co) under different conditions was evaluated by ELISA. Irradiated cells and unirradiated cells were both analyzed. (G) FCM analysis of PD-L1-targeting by NGHP in PANC-1 and PAN02 cells. NC, negative control. (H) ADCC assay was used to assess the functional status of αPD-L1 in NGHP. ADCC in PANC-1 and IR-PANC-1 (irradiated PANC-1) cells, mediated by different samples (“mNG” was mPEGS-nanogel), was induced using PBMC. All data are exhibited as the means ± SD (*n* = 3), and the inserted asterisks indicate statistically significant differences based on *P < 0.05, **P < 0.01, and ***P < 0.001.

Benefiting from the above excellent physicochemical properties and penetrability, it was hypothesized that the PFD@NGHP exhibited multifunctional biological therapeutic effect. As described in Fig. 5A, the Transwell assay system was designed to analyze the therapeutic effect of PFD@NGHP after penetration in mimicked ECM. Samples with the same initial concentration of PFD were added to the upper chamber, and after 48 h of incubation, different proliferation inhibitions across the 4 groups were observed in Fig. 5B. Unsatisfactorily, over 85% of hPSCs and mPSCs remained metabolically active after treatment with PFD alone, indicating a significant reduction in PFD concentration in the lower chamber due to the barrier effect of ECM. Similar to the PFD group, the mixture of PFD, HAase, and αPD-L1 was also hindered by ECM and just exhibited modest suppression toward hPSCs and mPSCs, with about 80% of the cells remaining viable. Differently, PFD@NGHP exhibited enhanced proliferation inhibition compared to the other groups, reducing the cell survival rate to 70%. This result demonstrated that the improved ECM penetration of PFD@NGHP led to a higher drug concentration in low chamber, which, in turn, resulted in a more significant inhibition of cell proliferation in both hPSCs and mPSCs. These results also suggested its ability to modulate the stroma via PSC suppression in PDAC.

By independently analyzing the response of each cell type to radiotherapy combined with PFD@NGHP therapy, the more comprehensive analysis of combination therapeutic efficacy across both tissue models and in vivo systems could be performed. Therefore, the proliferation inhibition by combination of PFD and radiotherapy was determined on PANC-1 cells, hPSCs, PAN02 cells, and mPSCs (Fig. 5C and Fig. S9A to E). As shown in Fig. S9A, after 48 h of incubation, a dose-dependent reduction in cellular viability was observed in cancer cells, which were more sensitive to radiotherapy compared to stellate cells. After combination with PFD or PFD@NGHP (0.3 mg/ml of PFD) and radiotherapy, 4 types of cells were all extremely inhibited (20% to 30% survival rate) after 10-Gy irradiation and PFD or PFD@NGHP treatment. Although PSCs, similar to fibroblast cells, exhibited moderate resistance to radiotherapy alone, a significantly enhanced suppression of cellular survival was observed when combining radiotherapy with PFD@NGHP (Fig. 5C and Fig. S9C and E). Although pancreatic cancer cells exhibited resistance to low doses of PFD or PFD@NGHP, the combined treatment with radiotherapy significantly enhanced the suppression of cellular viability. This possibly indicated that this approach, to some degree, was potential in cancer therapy, which was also a positive outcome (Fig. 5C and Fig. S9B and D). Moreover, the superior inhibitory efficacy of PFD@NGHP relative to PFD was hypothesized to result from enhanced PD-L1-mediated binding to cancer cells in this in vitro culture system. This interaction allowed for reductive disassembling and drug release directly at the cell surface, leading to a relatively higher drug concentration around the cancer cells. Importantly, this result also indicated that using PFD@NGHP during radiotherapy could enhance its inhibitory effects on PSCs, which would subsequently contribute to the suppression of fibrotic reactions and modulation of the tumor stroma (Fig. 5C and Fig. S9C and E).

Nevertheless, proliferation inhibition assays alone cannot conclusively verify the stromal amelioration of PFD@NGHP, necessitating further evaluation of suppression efficacy against activated fibroblast markers. Western blot was used to evaluate the expression of activated fibroblast markers, including collagen I, TGF-β1, and α-SMA, in both hPSCs and mPSCs. The results of Fig. 5D indicated that PFD and PFD@NGHP significantly down-regulated the expression of collagen I, TGF-β1, and α-SMA, which suggested that the encapsulated PFD maintained its pharmacological activity. The down-regulation of α-SMA, TGF-β1, and collagen I would significantly affect ECM production and stromal components, potentially alleviating desmoplasia [16]. Furthermore, the TGF-β1 down-regulation contributed to the reduced secretion of TGF-β1, which was also evaluated in coculture systems (Fig. 5E and F). As shown in Fig. 5E and F, NGHP, with its acceptable biocompatibility, caused minimal cellular damage and therefore did not affect TGF-β1 secretion levels. However, PFD and PFD@NGHP notably suppressed TGF-β1 secretion in both hPSCs and mPSCs (Fig. 5E and F). In the control group, PFD or PFD@NGHP reduced TGF-β1 secretion by about 50%, while in the radiotherapy group, the inhibitory effect was even more pronounced, reducing TGF-β1 secretion by approximately two-thirds. This enhanced suppression potentially resulted from the synergistic effect of PFD or PFD@NGHP in combination with radiotherapy on the monocultured PSCs. Meanwhile, the human/mouse coculture systems of PSCs and pancreatic cancer cells (H-Co and M-Co), which better mimicked the in vivo tumor stroma, were also applied to evaluate the inhibition of TGF-β1 secretion by PFD and PFD@NGHP. Compared to the monoculture system, the increased amplitude in TGF-β1 secretion induced by radiation was much higher in the coculture system. Both PFD and PFD@NGHP still effectively inhibited the secretion of TGF-β1 in the coculture system. However, the decreased amplitude of TGF-β1 was similar between the radiotherapy and control groups, with the inhibition level remaining at about 50%. The intercellular communication between the irradiated pancreatic cancer cells and irradiated PSCs might affect the TGF-β1 secretion, possibly via platelet-derived growth factor or integrin pathways [23,27]. These results might more accurately reflect the effect of PFD and PFD@NGHP in inhibiting TGF-β1 secretion in tumor stroma in vivo*.*

The potential of PFD@NGHP to ameliorate stromal fibrosis is noteworthy, while its ability to bind PD-L1 and rescue radiosensitivity represents a key rationale for combination therapy. The PD-L1-targeting ability of NGHP was assayed by flow cytometry in Fig. 5G. Regardless of whether it was PANC-1 or PAN02 cells, the PD-L1-targeting effect of NGHP was comparable to that of the αPD-L1 (avelumab), with the PD-L1 expression distribution exhibiting a rightward shift (Fig. 5G). It indicated that the synthesis of NGHP did not cause significant disruption to the antibody’s targeting function. Since avelumab is known for its ADCC effect, we further evaluated the ADCC activity induced by NGHP using PBMCs (effectors). PANC-1 and irradiated PANC-1 cells were the target cells. For some negative control groups of control, mPEGS-nanogel, and HAase, the nonspecific cell lysis did not exceed 5%, indicating the absence of ADCC effects (Fig. 5H). Due to the relatively low PD-L1 expression on conventional PANC-1 cells, less avelumab was bound, leading to moderate toxicity of about 7.5% cell lysis rate for simple antibody and NGHP. While in PANC-1 cells with PD-L1 up-regulation induced by radiation, the ADCC-induced cell lysis rate increased (Fig. 5H). Both avelumab and NGHP induced approximately 10% cell lysis, while nonspecific cell lysis remained below 5%, with a statistically significant difference. Therefore, avelumab in NGHP still retained its ability to induce effective ADCC, suggesting that its function was not impaired during the NGHP synthesis process. As a result, the PD-L1-targeting and ADCC-inducing functions of NGHP will play an important role in enhancing antitumor immune responses and rescuing radiosensitivity.

### Enhanced inhibition by radiotherapy and PFD@NGHP in organotypic 3D microtumors

**Fig. 6.**
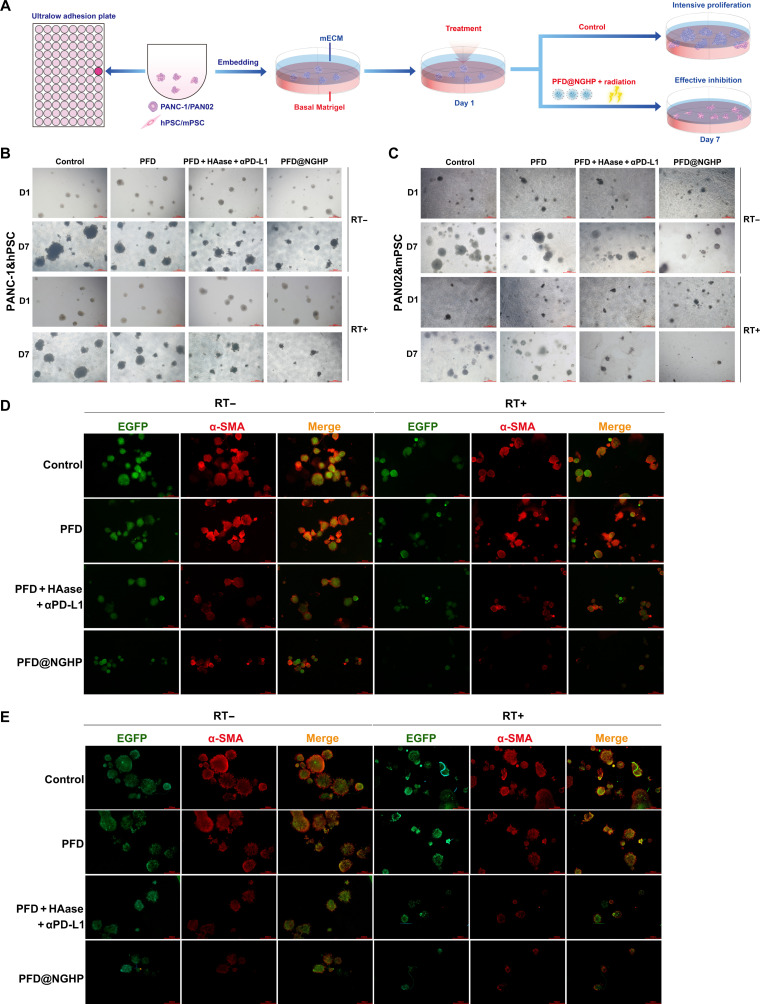
Radiotherapy synergized by PFD@NGHP assessed via colony formation of organotypic 3D microtumors. (A) Establishment schematic of organotypic 3D microtumors. (B) Inhibition of formation of organotypic 3D microtumors grown from PANC-1&hPSC seeds in bright field. On day 1 (D1), irradiated and unirradiated microtumors were both treated according to the predefined drug group combinations. On day 7, microtumors had grown to different sizes and were subsequently compared. (C) Bright-field images of organotypic 3D microtumors grown from PAN02&mPSC seeds, which resembled those in (B). (D) Immunofluorescence images of PANC-1&hPSC microtumors on day 7. PANC-1 cells and hPSCs in irradiated and unirradiated microtumors from different groups were distinguished by the expression of EGFP and α-SMA. Green fluorescence indicated the presence of PANC-1 cells expressing EGFP, while red fluorescence represented hPSCs expressing α-SMA. (E) Immunofluorescence images of PAN02&mPSC microtumors similar to (D). Green fluorescence indicated the PAN02 cells expressing EGFP, and red fluorescence indicated mPSCs expressing α-SMA. Scale bars, 500 μm.

Based on the excellent proliferation inhibition by combination of PFD@NGHP and radiotherapy in cellular level, we reconstructed the unique stromal architecture and desmoplastic microenvironment of PDAC in vitro to investigate changes in cellular composition following treatment. Given the complexity of the in vivo microenvironment, this method enabled more reliable prediction of stromal alterations posttreatment, particularly concerning cellular composition changes. Here, organotypic 3D microtumors derived from coculture system of PANC-1&hPSC or PAN02&mPSC that simulated stromal bioarchitecture of PDAC were cultured in Matrigel (Fig. 6A). After 5 d of coculture, the microtumor seeds (Fig. S10A and B) in ULA plates were transferred into the 3D culture Matrigel, followed by stabilization and addition of mimicked ECM. In addition, the initial state of microtumor seeds seeded in Matrigel was recorded 48 h after transplantation, which was exhibited in Fig. 6B “day 1” and Fig. 6C “day 1”. Subsequently, the spheroid-forming abilities under different conditions were compared. After 7 d of culture, the overview of PANC-1&hPSC and PAN02&mPSC microtumors in different groups were presented in Fig. 6B and C, respectively. In addition, the final immunofluorescence images of PANC-1&hPSC and PAN02&mPSC microtumors were presented in Fig. 6D and E, respectively. The quantitative analyses of spheroids count, volume, and fluorescence area ratio (FAR) in Fig. 6B to E were comprehensively presented in Fig. S11.

Clearly, with the advancement of therapeutic strategies, the in vitro colony formation ability of both PANC-1&hPSC and PAN02&mPSC microtumors exhibited a decreasing trend (Fig. 6B and C). This effect was chiefly characterized by declines in both numerical counts (Fig. S11A and D) and volumes (Fig. S11B and E) of spheroids. In both PANC-1&hPSC and PAN02&mPSC microtumors, the most abundant and largest spheroids were formed in the nonradiation control groups (Fig. 6B and C and Fig. S11A and D). In the radiation combined with PFD@NGHP group, the spheroids per field were the smallest and sparsest. In this group, some microtumors were successfully inhibited and remained at their initial size, while others shrank dramatically (Fig. 6B and C and Fig. S11B and E). In both types of microtumors, radiation achieved the limited inhibitory effects on clonogenicity in terms of both size and number. The ECM in the culture system hindered PFD penetration, leading to a significant reduction in PFD concentration and negligible inhibition to microtumor clonogenicity. The intragroup comparisons revealed that the PFD group and the PFD + HAase + αPD-L1 group did not exhibit effective inhibition of PANC-1&hPSC microtumors, despite exhibiting partial reduction of spheroids volume (Fig. 6B and Fig. S11A and B). Although the combined treatment (PFD + HAase + αPD-L1) was more effective than PFD alone under radiation conditions, it was still not as effective as PFD@NGHP. Consistent with prior results, a large amount of PFD@NGHP infiltrated through ECM by breaking HA barriers and facilitated PFD release in microtumors, finally synergizing the radiation. While Fig. 6C and Fig. S11D and E indicated superior suppression on clonogenicity by PFD or PFD + HAase + αPD-L1 in PAN02&mPSC microtumors relative to PANC-1&hPSC microtumors (likely attributable to heterogeneity), neither monotherapy nor RT-combinational therapy paradigms matched the potency of PFD@NGHP. The combination of PFD@NGHP and radiotherapy nearly eradicated the PAN02&mPSC microtumors. Overall, the significant shrinkage of 2 types of microtumors in the PFD@NGHP group strongly supported the notion that PFD@NGHP effectively penetrated in the microtumors and enhanced the radiotherapeutic efficiency, which demonstrated significant promise for in vivo applications.

As is known, the stroma of PDAC can occupy up to 90% of the entire tumor mass [16,17]. In addition, PSCs are the most prominent cell type in the PDAC stroma, constituting about 50% of it [45]. Therefore, it was important for us to evaluate the cellular composition changes in these posttherapy microtumors, which provided partial validation of stromal amelioration while predicting the therapeutic potential of PFD@NGHP in vivo. After evaluated by immunofluorescence, α-SMA proteins of hPSC and mPSC both emitted red fluorescence (Fig. S10C and D). As shown in Fig. 6D (PANC-1&hPSC microtumors) and Fig. 6E (PAN02&mPSC microtumors), the comparison of the size and number of microtumors among the groups yielded results consistent with the above findings in bright field. However, what deserved further attention was the variation in fluorescence intensity, which reflected the changes in the composition of cancer cells and PSCs within the microtumors. From the merged images in most groups (Fig. 6D and E), it was observed that the microtumors likely consisted of a green fluorescent core of cancer cells, with red fluorescence filling nearly the entire spheroid, indicating that the pancreatic cancer cells and PSCs were intimately mingled. These microtumors were similar to the PDAC tissues in vivo, where pancreatic cancer cells were surrounded by a large number of cancer-associated fibroblasts. When comparing the nonradiation groups of PANC-1&hPSC microtumors or PAN02&mPSC microtumors, we found that the red fluorescence in the combined treatment (PFD + HAase + αPD-L1) and PFD@NGHP groups was weaker than in the PFD and control groups, suggesting the inhibition of PSCs in both groups (Fig. 6D and E). In the radiation groups, the overall inhibitory effects on PSCs in microtumors seemed to be consistent with those observed in the nonradiation groups and were not intensively influenced by radiation, while the cancer cells in the microtumors gradually shrank after radiation. Interestingly, the fluorescence of the microtumors significantly weakened, and abnormal colocalization of fluorescence was observed in the PFD@NGHP group, indicating a disruption in the cellular composition of the microtumors and reflecting their terminal destiny after synergistic treatment. Obviously, changes in FAR suggested the impact on cellular composition by different treatments (Fig. S11C and F). With the advancement of therapeutic strategies, the FAR of both PANC-1&hPSC and PAN02&mPSC microtumors exhibited a similarly decreasing trend. According to the FAR value in Fig. S11C and F, neither single nor combined administration of PFD appeared to exert significant influence on cellular composition of microtumors. In both PFD + HAase + αPD-L1 group and PFD@NGHP group, a significant down-regulation of FAR values was observed, particularly under radiotherapy conditions, where levels decreased to near 1. However, without radiotherapy, only PFD@NGHP successfully reduced the FAR value to below 1, which indicated a significant decrease in the proportion of PSCs in microtumors. PSCs establish dynamic cross-talk with cancer cells, which collectively supports radioresistance via various inter- and intracellular pathways [23]. As evaluated in above experiments, PFD@NGHP successfully inhibited the proliferation of PSCs and their proportion in microtumors, which would disrupt their cross-talk with cancer cells and subsequently enhance radiosensitization of microtumors. Hence, PFD@NGHP’s versatility in delivering therapeutic agents further amplified its efficacy and modulated the cellular components of microtumors, which could be an innovative approach for overcoming traditional radiotherapy resistance of PDAC.

### In vivo antitumor effects and stromal amelioration

**Fig. 7.**
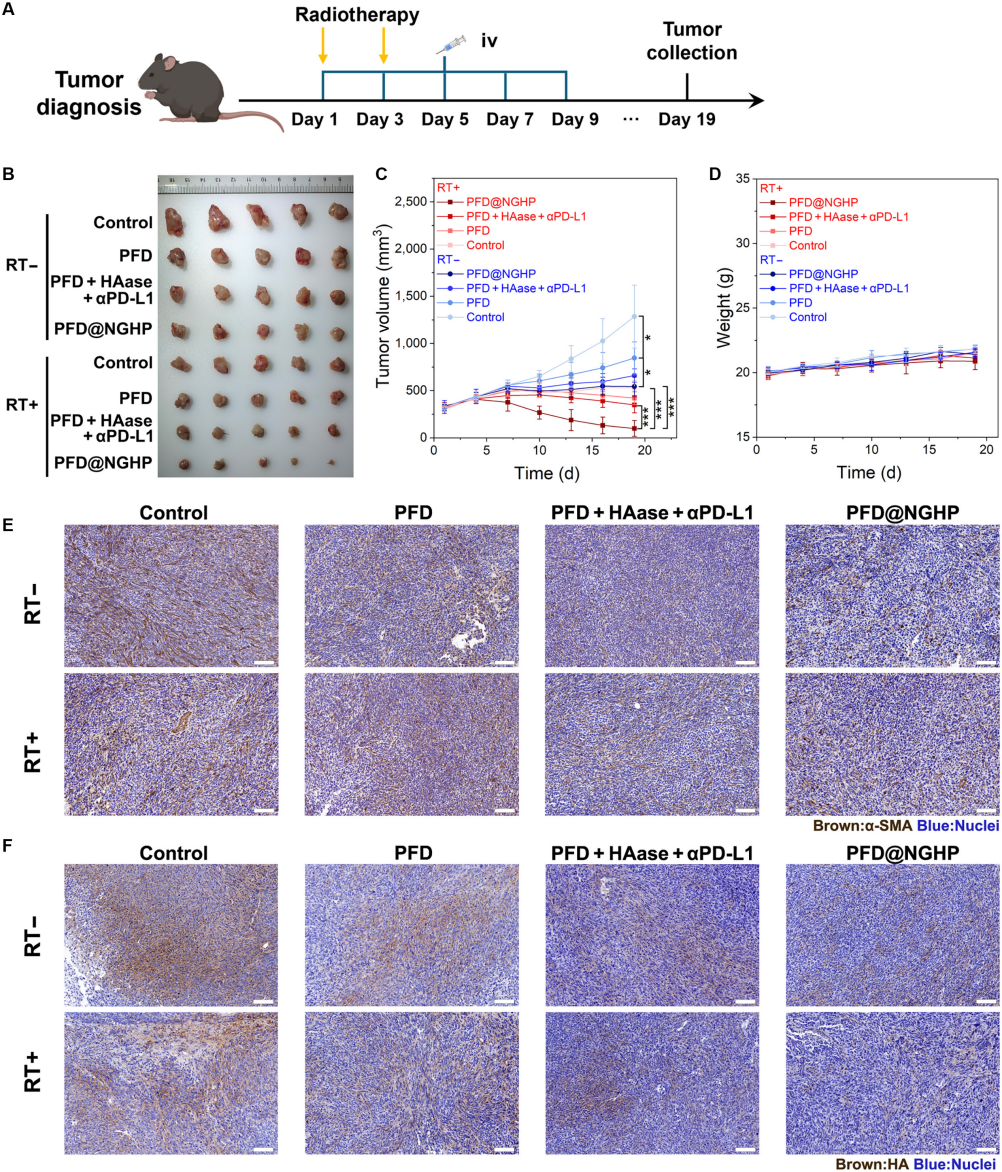
Evaluation of combinational therapy in mouse bearing tumor model. (A) Schematic of RT (6 Gy at day 1 and day 3), PFD (10 mg/kg), HAase (6.5 mg/kg), and αPD-L1 (avelumab; 12 mg/kg) were used via intravenous (iv) injection from day 1 to day 9 (qod) in PAN02 cells/mPSCs bearing mice. (B) Represent images of excised tumors after different treatments. (C) Tumor volume growth curves after different treatments. All data are exhibited as the means ± SD (*n* = 5), and the inserted asterisks indicate statistically significant differences based on *P < 0.05, **P < 0.01, and ***P < 0.001. (D) Body weight of tumor-bearing mice after different treatments. All data are exhibited as the means ± SD (*n* = 5). (E) Immunohistochemistry evaluation for α-SMA of PAN02&mPSC tumors obtained from C57BL/6 mice. (F) Immunohistochemistry evaluation for HA of PAN02&mPSC tumors obtained from C57BL/6 mice. Scale bars, 100 μm.

Motivated by impressive antitumor effect of PFD@NGHP in vitro, we pursued in vivo confirmation and therapeutic mechanism elucidation on C57BL/6 mouse model. Mice bearing pancreatic cancer were randomly administered tail vein injections of PBS, PFD, PFD + HAase + αPD-L1, and PFD@NGHP from day 1 to day 9 (qod), as shown in Fig. 7A. Radiation dose of 12 Gy/2 fractions was applied on day 1 and day 3. The final antitumor effects under different conditions are illustrated in Fig. 7B to D. The gross volume of excised tumors is almost consistent with the recorded volume measurements in 8 groups. In addition, the weight changes (Fig. 7D) in the mice exhibited that, for radiotherapy and nonradiotherapy groups, intragroup body weight differences were minimal, while intergroup differences were significant and notable. Unirradiated mice appeared to weigh more, likely due to the generally larger tumor size compared to irradiated mice. With radiation, PFD@NGHP exhibited the smallest tumor volume that was superior to PFD + HAase + αPD-L1 or PFD alone, indicating its significant antitumor effects. However, the antitumor effect would be significantly diminished when utilizing PFD@NGHP monotherapy, as it neither directly killed tumor cells nor induced tumor ICD. Collectively, the in vivo studies also validated the therapeutic synergy between PFD@NGHP and radiotherapy, achieving significant tumor eradication. In addition, histological examination of vital organs using HE staining demonstrated no significant damage in important organs of C57BL/6 treated in different groups (Fig. S12). This indicated that PFD@NGHP and radiotherapy exert negligible toxic effects on vital organs.

Furthermore, we examined the effect of various treatments on the PSCs population and activity (Fig. 7E and F and Fig. S13A and B). The area fraction representing these characteristic biomarker expressions in tumor tissues was semiquantitatively analyzed in Fig. S14. These results exhibited a progressive decrease (from about 50% to about 10%) in α-SMA (brown) expression levels in the tumor tissue as the treatment strategy improved (Fig. 7E and Fig. S14A). In the intragroup comparison under irradiated or unirradiated conditions, the PFD@NGHP group exhibited the lowest expression of α-SMA (about 10%), indicating the most significant inhibition of PSC activity (Fig. 7E and Fig. S14A). This is primarily attributed to the effective penetration of PFD@NGHP into the tumor, where it releases PFD to inhibit PSCs. No significant difference in α-SMA expression was observed between the radiotherapy and nonradiotherapy groups. Moreover, as shown in Figs. S13A and S14C, the immunofluorescence images of TGF-β1 expression in tumors also reflected the therapeutic efficacy of PFD@NGHP. Consistent with the results of the above study, radiation induced the high expression and secretion of TGF-β1 in tumors. Although PFD treatment alone provided only partial inhibition of TGF-β1 in tumor tissues, it significantly counteracted radiation-triggered TGF-β1 up-regulation (Fig. S14C). Unexpectedly, even PFD + HAase + αPD-L1 combination therapy resulted in similarly incomplete TGF-β1 inhibition as observed in PFD treatment. Remarkably, PFD@NGHP exhibited substantially enhanced inhibition of TGF-β1 in tumors. With residual expression reduced to about 8%, it outperformed all other therapeutic approaches (Fig. S14C). Regarding the abundance of ECM, including HA and collagen I, the immunohistochemistry results revealed that the PFD@NGHP group retained the lowest levels of both HA and collagen I within the irradiation and nonirradiation subgroups (Fig. 7E and Figs. S13B and S14B and D). In subgroup comparison, HA abundance of PFD@NGHP group was sparse and scattered, exhibiting an area fraction of about 10% under irradiation or nonirradiation conditions. While HA abundance of other groups was extensive and diffusive, including the combination treatment group (PFD + HAase + αPD-L1). A similar pattern was observed for collagen I, with significantly reduced amounts of intercellular collagen I in the PFD@NGHP group. Interestingly, no significant difference in collagen I expression was observed among PFD, PFD + HAase + αPD-L1, and PFD@NGHP groups, suggesting that PFD might exert moderate or indirect inhibitory effects on collagen I (Figs. S13B and S14D). PSCs were known to contribute to the production of ECM components, including collagen I and HA [23,45]. Therefore, the effective inhibition of PSCs and TGF-β1 could subsequently block the TGF-β1 secretion and probably suppress the production of HA and collagen I. Compared to the nonradiotherapy group, changes in the TME following radiotherapy might be a key factor contributing to alterations in the composition of ECM. Notably, in the irradiated PFD@NGHP group, the significant tumor regression indicated that the tumor had entered a terminal state, with its original structural composition already disrupted, probably leading to a reduction in HA and collagen I. These findings demonstrated the efficacy of PFD@NGHP in fundamentally ameliorating the tumor stroma, which improved treatment outcomes by restoring drug delivery efficiency and promoting radiosensitization.

### Evaluation of rescuing immunosuppression in vivo

**Fig. 8.**
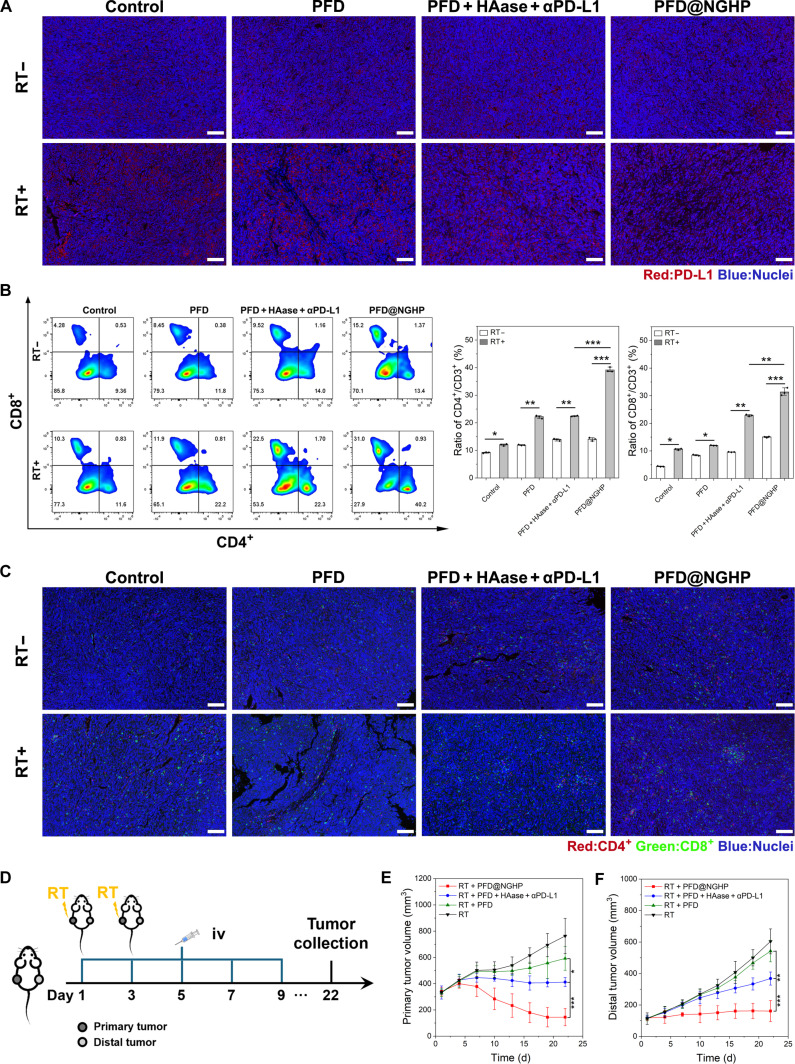
Evaluation of rescuing immunosuppression in mouse bearing tumor model. (A) Immunofluorescence images exhibited PD-L1 expression in PAN02&mPSC tumors after different treatments. Red fluorescence indicated PD-L1, and blue fluorescence indicated cellular nuclei. Scale bars, 100 μm. (B) FCM analysis of infiltrated T cells in PAN02&mPSC tumors. The intratumoral abundance of CD3^+^CD4^+^ T cells and CD3^+^CD8^+^ T cells were both assessed and compared. All data are exhibited as the means ± SD (*n* = 3), and the inserted asterisks indicate statistically significant differences based on *P < 0.05, **P < 0.01, and ***P < 0.001. (C) Immunofluorescence images exhibited T cell infiltration in PAN02&mPSC tumors after different treatments. Red fluorescence indicated CD3^+^CD4^+^ T cells, and green fluorescence indicated CD3^+^CD8^+^ T cells. The blue fluorescence represented cellular nuclei. Scale bars, 100 μm. (D) Schematic of monitoring abscopal responses to therapies in mice. Focal radiotherapy to primary tumors (6 Gy at day 1 and day 3). PFD (10 mg/kg), HAase (6.5 mg/kg), and αPD-L1 (avelumab; 12 mg/kg) were used via intravenous injection from day 1 to day 9 (qod) in PAN02 cell/mPSC-bearing mice. (E) The overall average primary tumor volume of each group and tumor growth of individual mouse after different treatments. (F) The overall average distal tumor volume of each group and tumor growth of individual mouse after different treatments. All data are exhibited as the means ± SD (*n* = 5), and the inserted asterisks indicate statistically significant differences based on *P < 0.05, **P < 0.01, and ***P < 0.001.

Clinically, a high tumor proportion score for PD-L1 expression and high immune cell infiltration in “hot” tumors were often associated with an improved response to PD-1/PD-L1 blockade therapies in various cancers [14]. Therefore, strategies to convert “cold” tumors into “hot” tumors are being vigorously explored to improve immunotherapy outcomes [8,31]. Despite pembrolizumab securing formal regulatory approval for the narrow subset (1% to 2%) of patients with pancreatic cancer harboring microsatellite instability-high or mismatch repair deficiency, the overwhelming majority remain nonresponsive to current PD-1/PD-L1 blockade therapies, constrained by the immunosuppressive TME of PDAC [5]. To evaluate the positive impact of the treatment combining PFD@NGHP with radiotherapy on the tumor immune microenvironment, we performed immunofluorescence and flow cytometry analysis. In flow cytometry analysis, the intratumoral CD4^+^ and CD8^+^ T cells were sorted according to gate strategy in Fig. S15. As illustrated in Fig. 8A and Fig. S16A, unirradiated tumors, including PFD@NGHP group, exhibited similarly low levels of PD-L1 expression. In general, the successful PD-L1 blockade would improve the immune surveillance via the classic immune response cycle, which contributed to the increased infiltration of immune cells and, in turn, induced the counterbalanced PD-L1 expression [10]. Since PFD@NGHP functions more like an immunological accelerant, it struggled to initiate a robust immune response cycle without radiotherapy serving as the “ignition”. However, through its dual mechanism of PD-L1 and TGF-β1 blockade, it probably enhanced immune cell infiltration or proliferation, as clearly demonstrated in Fig. 8B and C. Clinically, tumors with positive expression of PD-L1 were sensitive to PD-L1 blockade therapy, but its effect was weak in PDAC, correlating with a moderate antitumor response (tumors in Fig. 7B). Moreover, PD-L1 expression exhibited temporal and spatial variability during treatment, contributing to inconsistent therapeutic outcomes. When tumors were subjected to radiotherapy, a widespread up-regulation of PD-L1 expression was observed (Fig. 8A and Fig. S16A), which supported the therapeutic activity of αPD-L1. However, this up-regulation was effectively attenuated in the irradiated PFD@NGHP group. Based on the above experimental results, it was plausible that the TGF-β1 inhibition exerted a neutralizing effect against PD-L1 up-regulation, coupled with the therapeutic eradication of PD-L1-expressed tumor cells. Causally, with radiation, the dual-blockade effect of PFD@NGHP resulting in robust tumor regression exhibited in Fig. 7B and sparse cancer cells distribution in Fig. 8A.

Furthermore, the results of immunofluorescence and flow cytometry analysis in Fig. 8B and C and Fig. S16B and C validated the above speculation. According to the distribution of CD4^+^ and CD8^+^ T cells under unirradiated and irradiated conditions, radiotherapy significantly enhanced the infiltration of immune cells into the TME (Fig. 8B and Fig. S16B and C). In the unirradiated tumors, the percentages of CD4^+^ T cells were about 10% in the control group, 12% in the PFD group, 14% in the PFD + HAase + αPD-L1 group, and 14% in the PFD@NGHP group. In addition, intratumoral CD8^+^ T cell ratios were about 5% (control), 9% (PFD), 10% (PFD + HAase + αPD-L1), and 15% (PFD@NGHP). In the irradiated tumors, the infiltration of both CD4^+^ and CD8^+^ T cells was markedly enhanced. With radiotherapy, the PFD@NGHP group exhibited the most pronounced increase in the proportions of both CD4^+^ and CD8^+^ T cells. In the irradiated tumors, the CD4^+^ T cell ratios rose to approximately 12% in the control group, 22% in both the PFD and PFD + HAase + αPD-L1 groups, and 40% in the PFD@NGHP group. For CD8^+^ T cells, the percentages were around 10% (control), 11% (PFD), 23% (PFD + HAase + αPD-L1), and 31% (PFD@NGHP). Although spatial heterogeneity limited the representativeness of tumor-infiltrating T cell proportions assessed by histology, the distribution of intratumoral CD4^+^ and CD8^+^ T cells observed in immunofluorescence analysis was basically consistent with the above results of flow cytometry. Comparatively, PFD@NGHP group always generated the intense immune cell infiltration in both unirradiated and irradiated tumors (Fig. 8C and Fig. S16B and C). Obviously, Fig. 8C revealed some pronounced localized infiltration of immune cells in the irradiated PFD@NGHP group. This phenomenon not only demonstrated an activated immunogenic state but also reflected the distinct spatial heterogeneity in immune cell distribution. In general, the expression of PD-L1 and immune cell infiltration are closely interconnected in the TME, as confirmed by these findings. In addition, PFD@NGHP has been shown to exert beneficial regulatory effects on the periradiation immune microenvironment.

Radiotherapy has the potential to generate systemic tumor-targeting immune responses based on ICD, which is potential to eradicate anatomically distant, nonirradiated malignant lesions. This phenomenon is commonly referred to as “abscopal effect” [8,10]. To investigate whether this dual-blockade nanotherapy combined with radiotherapy elicited systemic antitumor immunity alongside tumor eradication, we used the bilateral tumor model to monitor the abscopal effect. Given our exclusive focus on the immunotherapeutic effects against distant tumors, we established primary tumors using PAN02 cells/mPSCs while developing distal tumors with PAN02 cells alone, based on our prior experimental findings. This approach was feasible to eliminate potential confounding influences from PFD treatment and stromal interactions on distant tumor suppression. As shown in Fig. 8E and F and Fig. S17, when combined with radiotherapy, both the PFD and PFD + HAase + αPD-L1 groups demonstrated partial inhibition of primary tumors. However, their disappointing control of distal tumors suggested their inability to induce potent abscopal effects (Fig. 8E and F). Noteworthily, PFD@NGHP coupled with radiotherapy not only achieved remarkable regression of primary tumors but also sustained long-term suppression of distal tumor progression, yielding pronounced therapeutic efficacy (Fig. 8E and F). These findings demonstrated a robust abscopal antitumor response elicited by PFD@NGHP in combination with radiotherapy. The promotion of the immune response cycle induced by radiotherapy was often counterbalanced by adaptive immune resistance in the TME, characterized by up-regulated PD-L1 expression [15]. However, the application of PFD@NGHP disrupted this balance by ablating the ECM barrier to facilitate TGF-β1/PD-L1 blockade and enhance immune cell infiltration. Consequently, tumor regression occurred through the combined effects of radiotherapy-induced damage and sustained immune-mediated cytotoxicity. These findings highlighted the potential of PFD@NGHP in combination with radiotherapy to optimize immunotherapy outcomes, presenting it as a promising strategy to enhance the clinical benefits of radiotherapy-induced adaptive immune response.

## Conclusion

In summary, this study developed the nanogel of PFD@NGHP to block the TGF-β1 and PD-L1 to synergize PDAC radiotherapy. The reduction-sensitive nanogel was tailored to effectively penetrate tumors and selectively disassemble in the TME, ensuring passive-targeting drug release. This was validated using advanced in vitro models including an orthotopic tumor in vitro culture model and organotypic 3D microtumors derived from coculture systems. These models accurately mimicked the desmoplastic stromal and spatial bioarchitecture of PDAC, providing reliable platforms for assessing the nanomedicine therapeutic potential. One of the standout features of PFD@NGHP is its ability to suppress TGF-β1 secretion and inhibit PSCs proliferation, ultimately ameliorating the stroma and down-regulating the PD-L1 expression (shown in Fig. 1). Another notable feature is its effective PD-L1 blockade capability, coupled with an additional ADCC effect, which plays a critical role in rescuing antitumor immunity (shown in Fig. 1). These 2 features are inherently interconnected and stepwise functionalization, designed explicitly to modulate the immunological and stromal changes in the irradiated TME. Thus, radiotherapy-induced counterbalanced immunosuppression environment will be further rescued by PFD@NGHP, resulting in robust tumor regression building on the stromal amelioration, cancer cell death, and immune response. In our study, this integrated functional nanocarrier of PFD@NGHP emerges as a promising strategy for optimizing therapeutic outcomes in PDAC radiotherapy. However, to translate these preclinical results into clinical applications, scaling up PFD@NGHP poses critical challenges. During good-manufacturing-practice-compliant production, ensuring batch uniformity and optimal nanogel size control is crucial. Long-term stability further requires mitigation of payload leakage and particle aggregation, necessitating optimized lyophilization protocols and standardized storage conditions [46]. Moreover, the systemic immunogenicity induced by exogenous nanocomponents also requires concern. As is known, PEI has been widely used to deliver vaccines or adjuvants to robustly stimulate immune responses [47]. For instance, the PEI-based nanoadjuvant Bo-112—administered intratumorally in patients with cancer—demonstrated potent antitumor activity by activating immune responses [48]. However, PEI clinical utility may be limited because of cationic-charge-driven inflammatory side effects, and how to use and administer it remains the key challenge [49]. To mitigate these issues, surface shielding strategies (e.g., PEGylation or human-derived protein modification) can be used [49]. rHuPH20, a human-derived HAase, shows low immunogenicity clinically [23,50]. The HALO 202 trial demonstrated that combining it with gemcitabine significantly enhanced antitumor efficacy against pancreatic cancer [50]. In addition, this also warrants future improvements in our work of HAase application. Finally, the synthetic strategy, detailed characterization, and comprehensive evaluation using advanced 3D tumor models in this study are expected to provide valuable insights into clinical translatability.

## Ethical Approval

The authors state that animal experiments were conducted with the approval and guidance of the Ethics Committee of Animal Care and Use of Naval Medical University.

## Data Availability

All data generated or analyzed during this study are included in this article.

## References

[1] Siegel RL, Giaquinto AN, Jemal A. Cancer statistics. CA Cancer J Clin. 2024;74(1):12–49. 38230766 10.3322/caac.21820

[2] Halbrook CJ, Lyssiotis CA, Pasca di Magliano M, Maitra A. Pancreatic cancer: Advances and challenges. Cell. 2023;186(8):1729–1754. 37059070 10.1016/j.cell.2023.02.014 PMC10182830

[3] Hu ZI, O’Reilly EM. Therapeutic developments in pancreatic cancer. Nat Rev Gastroenterol Hepatol. 2024;21(1):7–24. 37798442 10.1038/s41575-023-00840-w

[4] Shi Y, Wang Y, Zhang W, Niu K, Mao X, Feng K, Zhang Y. N6-methyladenosine with immune infiltration and PD-L1 in hepatocellular carcinoma: Novel perspective to personalized diagnosis and treatment. Front Endocrinol. 2023;14:1153802. 10.3389/fendo.2023.1153802 PMC10352105 37469973

[5] Marabelle A, Le DT, Ascierto PA, Di Giacomo AM, De Jesus-Acosta A, Delord JP, Geva R, Gottfried M, Penel N, Hansen AR, et al. Efficacy of pembrolizumab in patients with noncolorectal high microsatellite instability/mismatch repair-deficient cancer: Results from the phase II KEYNOTE-158 study. J Clin Oncol. 2020;38(1):1–10. 31682550 10.1200/JCO.19.02105 PMC8184060

[6] Mizrahi O, Michaeli TF, Hubert A, Wygoda MR, Blumenfeld P. Stereotactic body radiotherapy compared to conventionally fractionated radiotherapy for locally advanced or oligometastatic pancreatic cancer. J Clin Oncol. 2024;42(3):677–677.

[7] Zhu X, Chen D, Cao Y, Zhao X, Ju X, Shen Y, Cao F, Qing S, Fang F, Jia Z, et al. Validation of the eighth edition of the AJCC staging system for patients with pancreatic adenocarcinoma initially receiving chemoradiotherapy and proposal of modifications. Cancer Biol Med. 2020;17(2):492–500. 32587784 10.20892/j.issn.2095-3941.2019.0101 PMC7309473

[8] Lynch C, Pitroda SP, Weichselbaum RR. Radiotherapy, immunity, and immune checkpoint inhibitors. Lancet Oncol. 2024;25(8):E352–E362. 39089313 10.1016/S1470-2045(24)00075-5

[9] Barker HE, Paget JT, Khan AA, Harrington KJ. The tumour microenvironment after radiotherapy: Mechanisms of resistance and recurrence. Nat Rev Cancer. 2015;15(7):409–425. 26105538 10.1038/nrc3958 PMC4896389

[10] Kroemer G, Galassi C, Zitvogel L, Galluzzi L. Immunogenic cell stress and death. Nat Immunol. 2022;23(4):487–500. 35145297 10.1038/s41590-022-01132-2

[11] Mills BN, Qiu H, Drage MG, Chen C, Mathew JS, Garrett-Larsen J, Ye J, Uccello TP, Murphy JD, Belt BA, et al. Modulation of the human pancreatic ductal adenocarcinoma immune microenvironment by stereotactic body radiotherapy. Clin Cancer Res. 2022;28(1):150–162. 34862242 10.1158/1078-0432.CCR-21-2495 PMC8738140

[12] Sato H, Niimi A, Yasuhara T, Permata TBM, Hagiwara Y, Isono M, Nuryadi E, Sekine R, Oike T, Kakoti S, et al. DNA double-strand break repair pathway regulates PD-L1 expression in cancer cells. Nat Commun. 2017;8(1):1751. 29170499 10.1038/s41467-017-01883-9 PMC5701012

[13] Sun LL, Yang RY, Li CW, Chen MK, Shao B, Hsu JM, Chan LC, Yang Y, Hsu JL, Lai YJ, et al. Inhibition of ATR downregulates PD-L1 and sensitizes tumor cells to T cell-mediated killing. Am J Cancer Res. 2018;8(7):1307–1316. 30094103 PMC6079156

[14] Kornepati AVR, Vadlamudi RK, Curiel TJ. Programmed death ligand 1 signals in cancer cells. Nat Rev Cancer. 2022;22(3):174–189. 35031777 10.1038/s41568-021-00431-4 PMC9989967

[15] Yoon HH, Jin Z, Kour O, Kankeu Fonkoua LA, Shitara K, Gibson MK, Prokop LJ, Moehler M, Kang YK, Shi Q, et al. Association of PD-L1 expression and other variables with benefit from immune checkpoint inhibition in advanced gastroesophageal cancer: Systematic review and meta-analysis of 17 phase 3 randomized clinical trials. JAMA Oncol. 2022;8(10):1456–1465. 36006624 10.1001/jamaoncol.2022.3707 PMC9412834

[16] Valkenburg KC, de Groot AE, Pienta KJ. Targeting the tumour stroma to improve cancer therapy. Nat Rev Clin Oncol. 2018;15(6):366–381. 29651130 10.1038/s41571-018-0007-1 PMC5960434

[17] Ho WJ, Jaffee EM, Zheng L. The tumour microenvironment in pancreatic cancer - Clinical challenges and opportunities. Nat Rev Clin Oncol. 2020;17(9):527–540. 32398706 10.1038/s41571-020-0363-5 PMC7442729

[18] Farhood B, Khodamoradi E, Hoseini-Ghahfarokhi M, Motevaseli E, Mirtavoos-Mahyari H, Eleojo Musa A, Najafi M. TGF-β in radiotherapy: Mechanisms of tumor resistance and normal tissues injury. Pharmacol Res. 2020;155: Article 104745. 32145401 10.1016/j.phrs.2020.104745

[19] Zhang Y, Lv N, Li M, Liu M, Wu C. Cancer-associated fibroblasts: Tumor defenders in radiation therapy. Cell Death Dis. 2023;14(8):541. 37607935 10.1038/s41419-023-06060-z PMC10444767

[20] Messeha SS, Zarmouh NO, Soliman KFA. Polyphenols modulating effects of PD-L1/PD-1 checkpoint and EMT-mediated PD-L1 overexpression in breast cancer. Nutrients. 2021;13(5):1718. 34069461 10.3390/nu13051718 PMC8159140

[21] Pan LN, Ma YF, Li Z, Hu JA, Xu ZH. KRAS G12V mutation upregulates PD-L1 expression via TGF-β/EMT signaling pathway in human non-small-cell lung cancer. Cell Biol Int. 2021;45(4):795–803. 33325140 10.1002/cbin.11524

[22] Xia Q, Jia J, Hu C, Lu J, Li J, Xu H, Fang J, Feng D, Wang L, Chen Y. Tumor-associated macrophages promote PD-L1 expression in tumor cells by regulating PKM2 nuclear translocation in pancreatic ductal adenocarcinoma. Oncogene. 2022;41(6):865–877. 34862460 10.1038/s41388-021-02133-5 PMC8816727

[23] Schnittert J, Bansal R, Prakash J. Targeting pancreatic stellate cells in cancer. Trends Cancer. 2019;5(2):128–142. 30755305 10.1016/j.trecan.2019.01.001

[24] Chang CH, Pauklin S. ROS and TGFβ: From pancreatic tumour growth to metastasis. J Exp Clin Cancer Res. 2021;40(1):152. 33941245 10.1186/s13046-021-01960-4 PMC8091747

[25] Richter K, Konzack A, Pihlajaniemi T, Heljasvaara R, Kietzmann T. Redox-fibrosis: Impact of TGFβ1 on ROS generators, mediators and functional consequences. Redox Biol. 2015;6:344–352. 26335400 10.1016/j.redox.2015.08.015 PMC4565043

[26] Gulley JL, Schlom J, Barcellos-Hoff MH, Wang XJ, Seoane J, Audhuy F, Lan Y, Dussault I, Moustakas A. Dual inhibition of TGF-β and PD-L1: A novel approach to cancer treatment. Mol Oncol. 2022;16(11):2117–2134. 34854206 10.1002/1878-0261.13146 PMC9168966

[27] Deng Z, Fan T, Xiao C, Tian H, Zheng Y, Li C, He J. TGF-β signaling in health, disease, and therapeutics. Signal Transduct Target Ther. 2024;9(1):61. 38514615 10.1038/s41392-024-01764-w PMC10958066

[28] Lei Y, Xu J, Xiao M, Wu D, Xu H, Yang J, Mao X, Pan H, Yu X, Shi S. Pirfenidone alleviates fibrosis by acting on tumour-stroma interplay in pancreatic cancer. Br J Cancer. 2024;130(9):1505–1516. 38454166 10.1038/s41416-024-02631-9 PMC11058874

[29] Kozono S, Ohuchida K, Eguchi D, Ikenaga N, Fujiwara K, Cui L, Mizumoto K, Tanaka M. Pirfenidone inhibits pancreatic cancer desmoplasia by regulating stellate cells. Cancer Res. 2013;73(7):2345–2356. 23348422 10.1158/0008-5472.CAN-12-3180

[30] Mpekris F, Papaphilippou PC, Panagi M, Voutouri C, Michael C, Charalambous A, Marinov Dinev M, Katsioloudi A, Prokopi-Demetriades M, Anayiotos A, et al. Pirfenidone-loaded polymeric micelles as an effective mechanotherapeutic to potentiate immunotherapy in mouse tumor models. ACS Nano. 2023;17(24):24654–24667. 38054429 10.1021/acsnano.3c03305 PMC10753878

[31] Lan Y, Moustafa M, Knoll M, Xu C, Furkel J, Lazorchak A, Yeung TL, Hasheminasab SM, Jenkins MH, Meister S, et al. Simultaneous targeting of TGF-β/PD-L1 synergizes with radiotherapy by reprogramming the tumor microenvironment to overcome immune evasion. Cancer Cell. 2021;39(10):1388–1403.e10. 34506739 10.1016/j.ccell.2021.08.008

[32] Tapia-Galisteo A, Sanchez-Rodriguez I, Narbona J, Iglesias-Hernandez P, Aragon-Garcia S, Jimenez-Reinoso A, Compte M, Khan S, Tsuda T, Chames P, et al. Combination of T cell-redirecting strategies with a bispecific antibody blocking TGF-β and PD-L1 enhances antitumor responses. Onco Targets Ther. 2024;13(1):2338558. 10.1080/2162402X.2024.2338558 PMC11018002 38623463

[33] Li W, Li J, Gao J, Li B, Xia Y, Meng Y, Yu Y, Chen H, Dai J, Wang H, et al. The fine-tuning of thermosensitive and degradable polymer micelles for enhancing intracellular uptake and drug release in tumors. Biomaterials. 2011;32(15):3832–3844. 21377724 10.1016/j.biomaterials.2011.01.075

[34] Chen D, Zhu X, Tao W, Kong Y, Huag Y, Zhang Y, Liu R, Jiang L, Tang Y, Yu H, et al. Regulation of pancreatic cancer microenvironment by an intelligent gemcitabine@nanogel system via in vitro 3D model for promoting therapeutic efficiency. J Control Release. 2020;324:545–559. 32504777 10.1016/j.jconrel.2020.06.001

[35] Li W, Zhao H, Qian W, Li H, Zhang L, Ye Z, Zhang G, Xia M, Li J, Gao J, et al. Chemotherapy for gastric cancer by finely tailoring anti-Her2 anchored dual targeting immunomicelles. Biomaterials. 2012;33(21):5349–5362. 22542611 10.1016/j.biomaterials.2012.04.016

[36] Javanmardi S, Tamaddon AM, Aghamaali MR, Ghahramani L, Abolmaali SS. Redox-sensitive, PEG-shielded carboxymethyl PEI nanogels silencing microRNA-21, sensitizes resistant ovarian cancer cells to cisplatin. Asian J Pharm Sci. 2020;15(1):69–82. 32175019 10.1016/j.ajps.2018.10.006 PMC7066047

[37] Zhu X, Sun Y, Chen D, Li J, Dong X, Wang J, Chen H, Wang Y, Zhang F, Dai J, et al. Mastocarcinoma therapy synergistically promoted by lysosome dependent apoptosis specifically evoked by 5-Fu@nanogel system with passive targeting and pH activatable dual function. J Control Release. 2017;254:107–118. 28342982 10.1016/j.jconrel.2017.03.038

[38] Parmar VK, Desai SB, Vaja T. RP-HPLC and UV spectrophotometric methods for estimation of pirfenidone in pharmaceutical formulations. Indian J Pharm Sci. 2014;76(3):225–229. 25035534 PMC4090830

[39] Conte C, Mastrotto F, Taresco V, Tchoryk A, Quaglia F, Stolnik S, Alexander C. Enhanced uptake in 2D- and 3D- lung cancer cell models of redox responsive PEGylated nanoparticles with sensitivity to reducing extra- and intracellular environments. J Control Release. 2018;277:126–141. 29534890 10.1016/j.jconrel.2018.03.011

[40] Chaiswing L, Oberley TD. Extracellular/microenvironmental redox state. Antioxid Redox Signal. 2010;13(4):449–465. 20017602 10.1089/ars.2009.3020

[41] Chaiswing L, Zhong W, Cullen JJ, Oberley LW, Oberley TD. Extracellular redox state regulates features associated with prostate cancer cell invasion. Cancer Res. 2008;68(14):5820–5826. 18632636 10.1158/0008-5472.CAN-08-0162

[42] Arodin Selenius L, Wallenberg Lundgren M, Jawad R, Danielsson O, Bjornstedt M. The cell culture medium affects growth, phenotype expression and the response to selenium cytotoxicity in A549 and HepG2 cells. Antioxidants. 2019;8(5):130. 31091728 10.3390/antiox8050130 PMC6563005

[43] Sun W, Davis PB. Reducible DNA nanoparticles enhance in vitro gene transfer via an extracellular mechanism. J Control Release. 2010;146(1):118–127. 20438780 10.1016/j.jconrel.2010.04.031 PMC3031909

[44] Chen D, Wang J, Wang Y, Zhang F, Dong X, Jiang L, Tang Y, Zhang H, Li W. Promoting inter-/intra-cellular process of nanomedicine through its physicochemical properties optimization. Curr Drug Metab. 2018;19(1):75–82. 29268683 10.2174/1389200219666171221122119

[45] Apte MV, Wilson JS, Lugea A, Pandol SJ. A starring role for stellate cells in the pancreatic cancer microenvironment. Gastroenterology. 2013;144(6):1210–1219. 23622130 10.1053/j.gastro.2012.11.037 PMC3729446

[46] Zhang X, Chan HW, Shao Z, Wang Q, Chow S, Chow SF. Navigating translational research in nanomedicine: A strategic guide to formulation and manufacturing. Int J Pharm. 2025;671: Article 125202. 39799998 10.1016/j.ijpharm.2025.125202

[47] Hafner AM, Corthesy B, Merkle HP. Particulate formulations for the delivery of poly(I:C) as vaccine adjuvant. Adv Drug Deliv Rev. 2013;65(10):1386–1399. 23751781 10.1016/j.addr.2013.05.013

[48] Aznar MA, Planelles L, Perez-Olivares M, Molina C, Garasa S, Etxeberria I, Perez G, Rodriguez I, Bolanos E, Lopez-Casas P, et al. Immunotherapeutic effects of intratumoral nanoplexed poly I:C. J Immunother Cancer. 2019;7(1):116. 31046839 10.1186/s40425-019-0568-2 PMC6498680

[49] Chilukuri N, Sun W, Parikh K, Naik RS, Tang L, Doctor BP, Saxena A. A repeated injection of polyethyleneglycol-conjugated recombinant human butyrylcholinesterase elicits immune response in mice. Toxicol Appl Pharmacol. 2008;231(3):423–429. 18586293 10.1016/j.taap.2008.05.016

[50] Hingorani SR, Zheng L, Bullock AJ, Seery TE, Harris WP, Sigal DS, Braiteh F, Ritch PS, Zalupski MM, Bahary N, et al. HALO 202: Randomized phase II study of PEGPH20 plus nab-paclitaxel/gemcitabine versus nab-paclitaxel/gemcitabine in patients with untreated, metastatic pancreatic ductal adenocarcinoma. J Clin Oncol. 2018;36(4):359–366. 29232172 10.1200/JCO.2017.74.9564

