# Supplementary material for: Rescue Radiosensitization of Pancreatic Cancer via PD-L1/TGF-β1 Dual-Blockade Nanotherapy as Evaluated in 3-Dimensional Microtumors
Source: Biomater Res. 2026 Mar 4;30:0335. doi: 10.34133/bmr.0335 (PMC12957541; doi:10.34133/bmr.0335)

**Supplementary Figure 1: TGF-β1 in supernatant of pancreatic cancer cells**

TGF-β1 secretion by pancreatic cancer cells under different conditions was quantified using ELISA. The levels remained low under different conditions, and following PFD treatment, TGF-β1 levels further decreased. As a result, these low concentrations likely had minimal impact on the detection values in the co-culture system. All data are exhibited as the mean ± SD (n = 3).


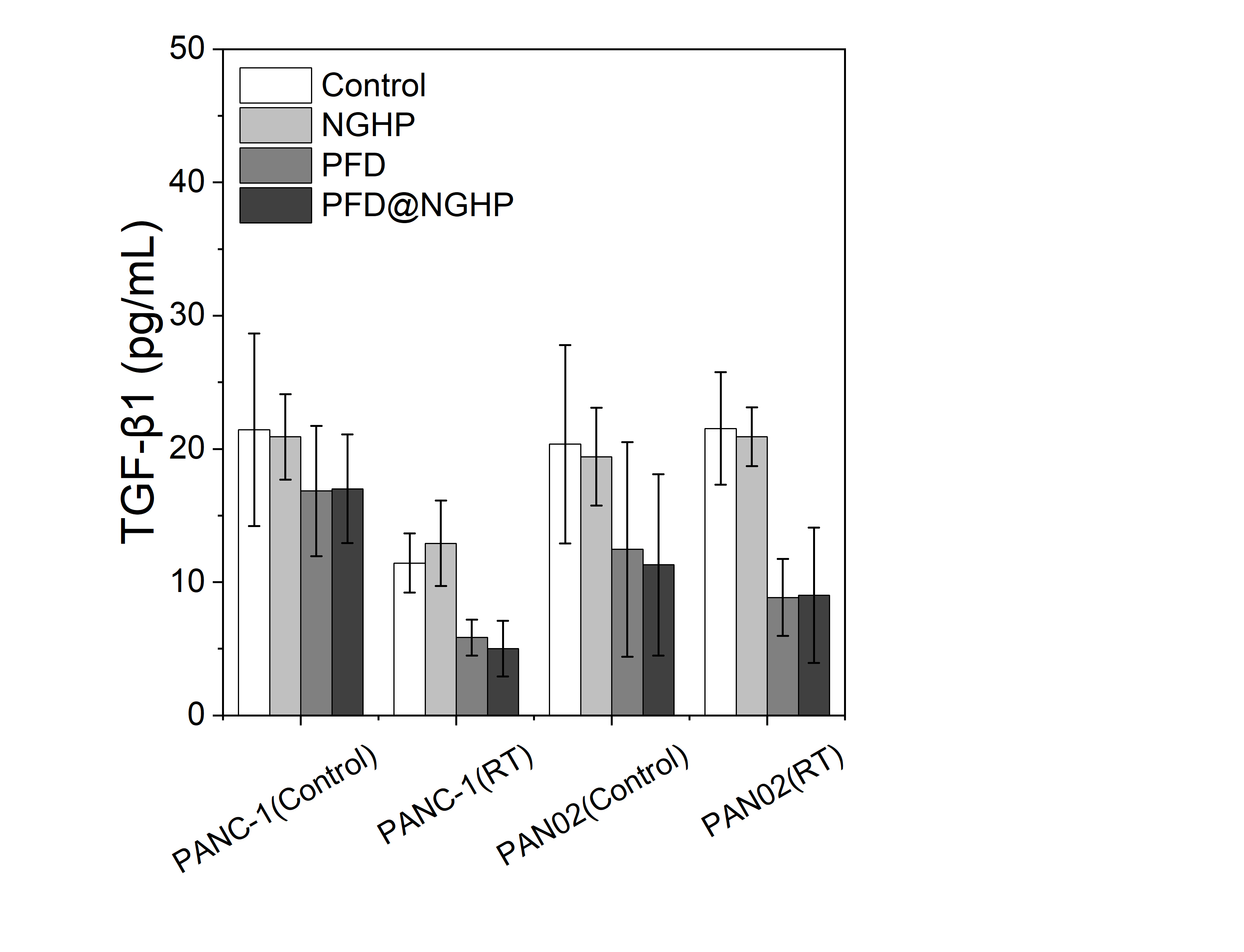


**Supplementary Figure 2: PD-L1 expression in tumor tissues**

The area fraction of PD-L1 expression of tumor tissues was quantified from immunofluorescence images in Figure 2E*.* All data are exhibited as the mean ± SD (n = 3), and the inserted asterisks indicate statistically significant differences based on *p* < 0.05(*), *p* < 0.01(**) and *p* < 0.001(***).


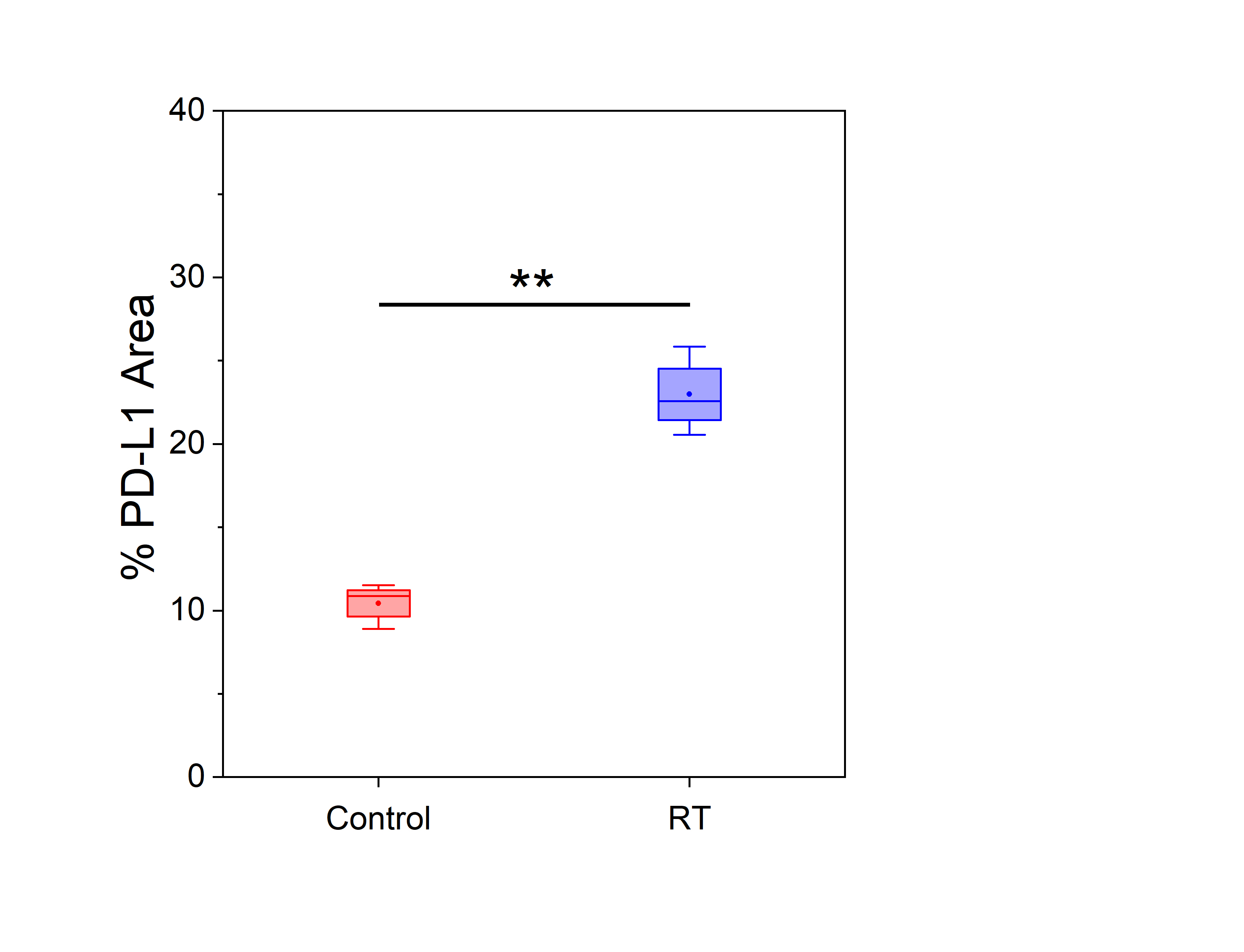


**Supplementary Figure 3: Mean fluorescence intensity of PD-L1 expression in different conditions**

Mean fluorescence intensity derived from the FCM histograms of Figure 2F for comparisons of PD-L1 expression in different conditions. All data are exhibited as the mean ± SD (n = 3), and the inserted asterisks indicate statistically significant differences based on *p* < 0.05(*), *p* < 0.01(**) and *p* < 0.001(***).


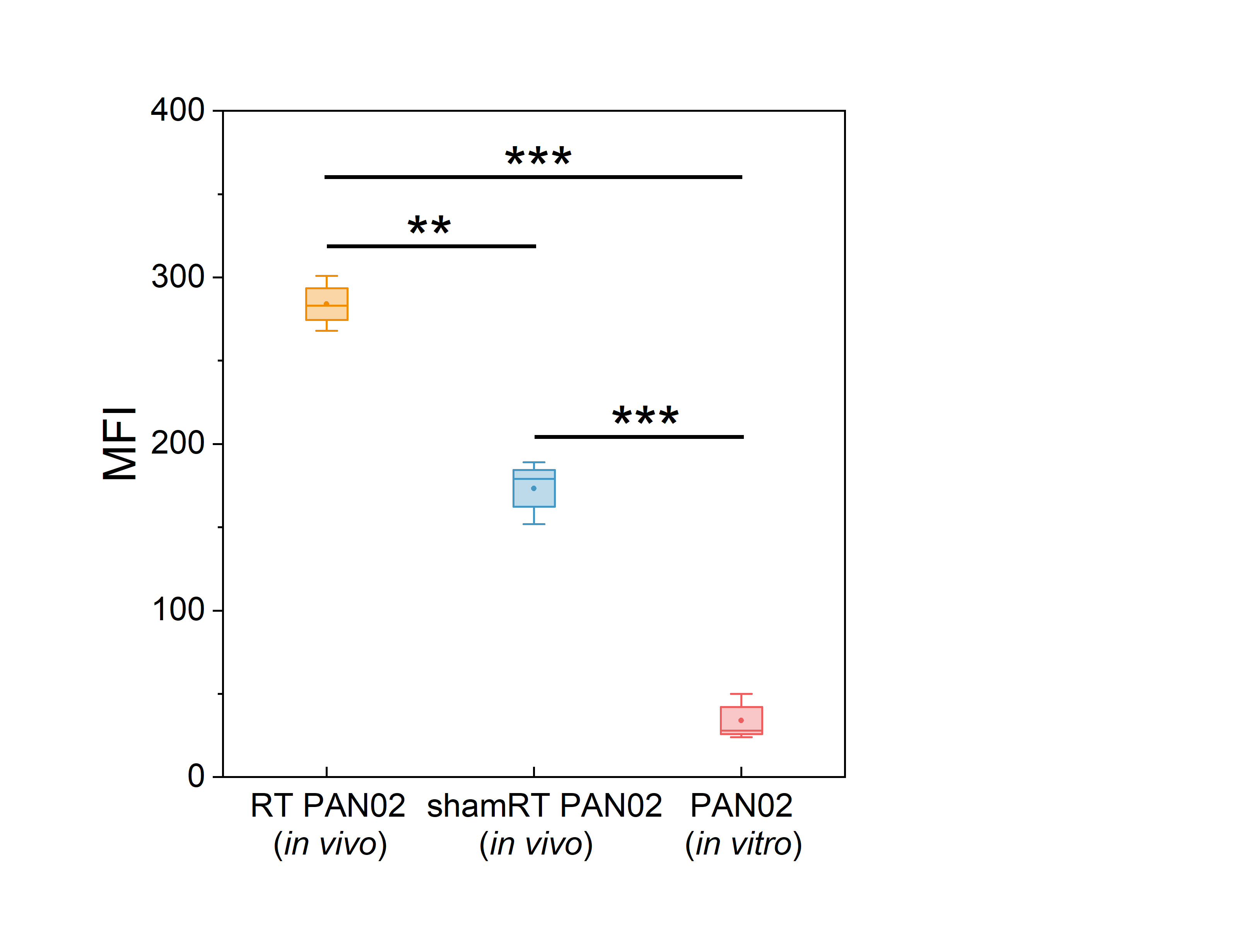


**Supplementary Figure 4:** The calibration curves generated from standard L-glycine solutions (A) and bPEI solutions (B) via TNBS assay were used to determine the consumption of primary amino groups of PEI nanogels. All data are exhibited as the mean ± SD (n = 3).


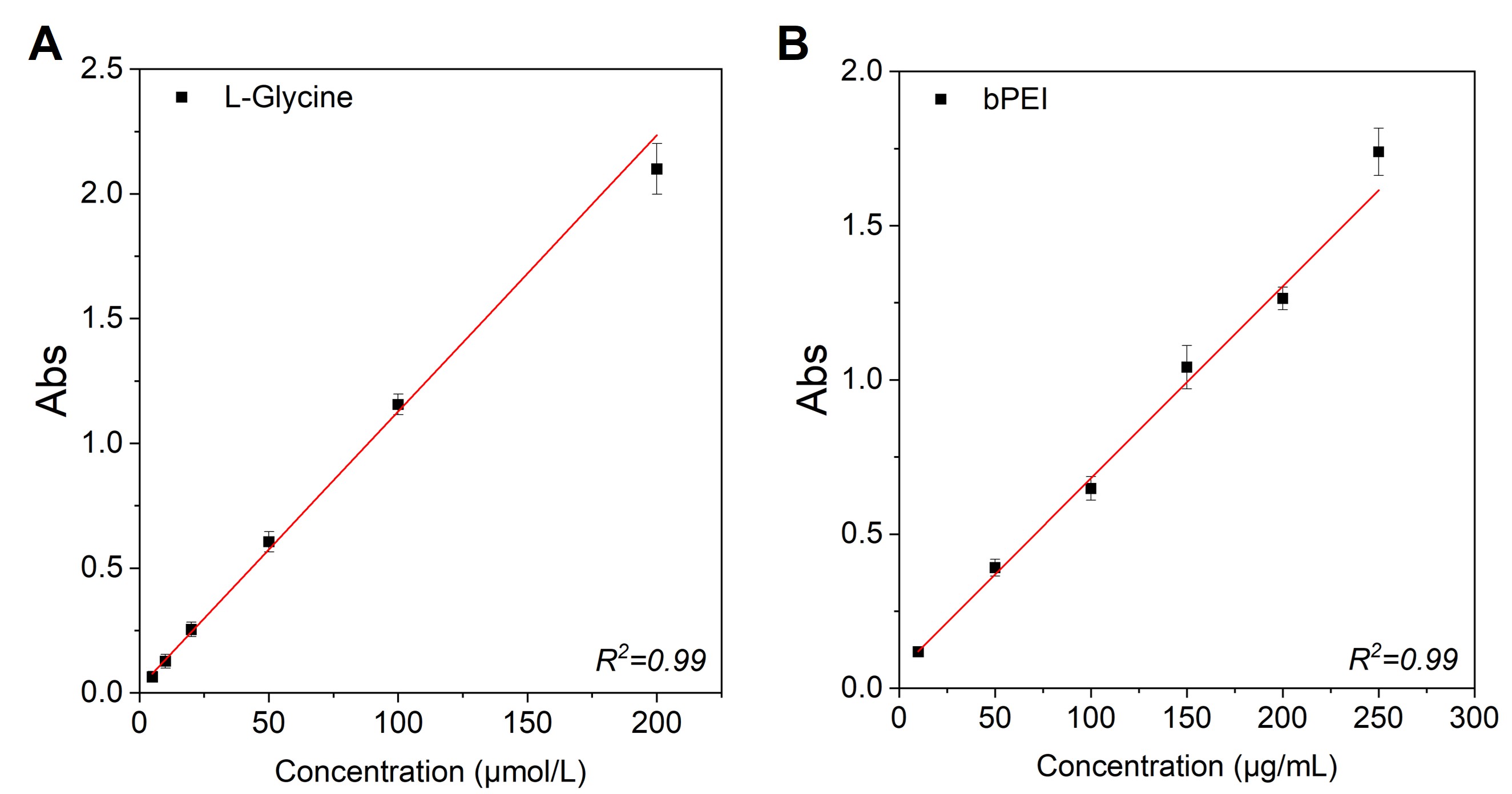


**Supplementary Figure 5:** (A) Size distribution in conditioned DMEM medium from 0 h to 96 h. (B) Berry plot of the SLLS data tested from the NGHP

A range of nanogel concentrations were prepared by weighting method. Static laser light scattering (SLLS) was conducted by the ALV CG-3, the measurements of the nanogels were implemented by varying the scattering angles from 30° to 150°, with an increase of 5° stepwise. Three concentrations (Nanogel: 0.208, 0.277 and 0.333 mg/mL) of each simple were prepared and the Berry method (the square root of the expression used in the Zimm method) was used in SLLS experiment:

$\sqrt{\frac{Kc}{R}}=\sqrt{\frac{1}{M_{w}}(1+\frac{1}{3}{R_{g}}^{2}q^{2})+2A_{2}c}$

where *K*, *c*, *R*, *M_w_*, *q*, *R_g_,* and *A_2_* are the Rayleigh ratio at a specific measurement, nanogel concentration, the refractive index of solvent, molecular weight, angle, radius of gyration, and the second virial coefficient, respectively. Based on these experimental data, dotted lines in these two plots were obtained by extrapolating both angles and concentrations to zero. And the intercept of the dotted line is $\sqrt{\frac{1}{M_{w}}}$ which can obtain molar mass of the nanoparticle. Similarly, *R_g_* was obtained by extrapolating angles to zero and *A_2_* was obtained by extrapolating concentrations to zero. Additional, in this experiment, the particle size ratio as shown in following function is an important parameter that qualitatively reflects the structural information of the particles. *ρ (R_g_/R_h_)*

where *R_g_* and *R_h_* is the average radius of gyration and the hydrodynamic radius acquired from static and dynamic measurements respectively.


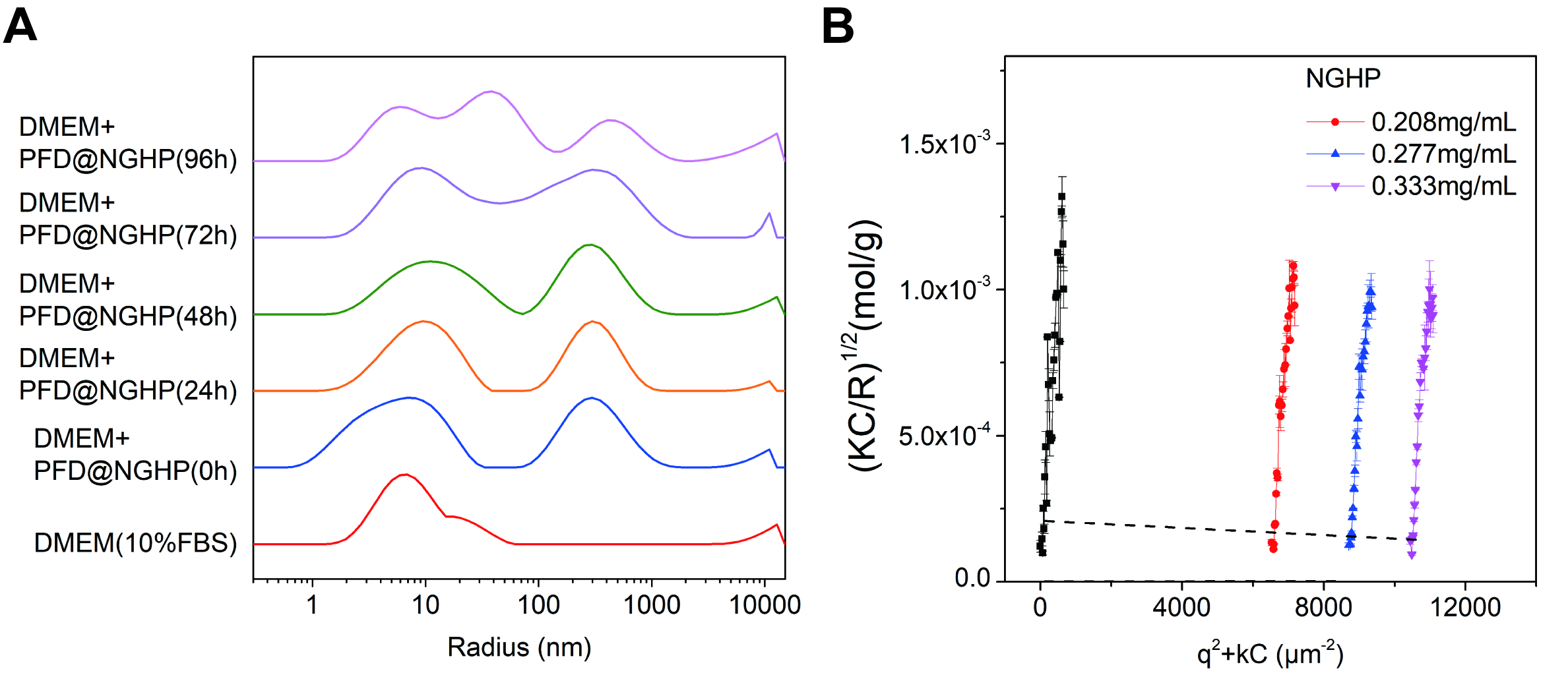


**Supplementary Figure 6:** (A) Representative UV absorption spectrum for the pirfenidone (25 μg/mL). (B) Calibration curve of concentration for pirfenidone was obtained from standard solutions by UV spectrophotometer. (C) Representative UV absorption spectrum for PFD (100 μg/mL), NGHP and PFD@NGHP.





**Supplementary Figure 7: Biocompatibility of NGHP and its component in hTERT-HPNE cells.**

Similar to other cells, the mPEGS modified nanogels exhibited certain toxicity to normal human pancreatic ductal epithelial cells at high concentrations, but NGHP exhibited significantly reduced toxicity toward these cells, which also posed relatively lower risks to normal pancreatic tissues. All data are exhibited as the mean ± SD (n = 3).

**
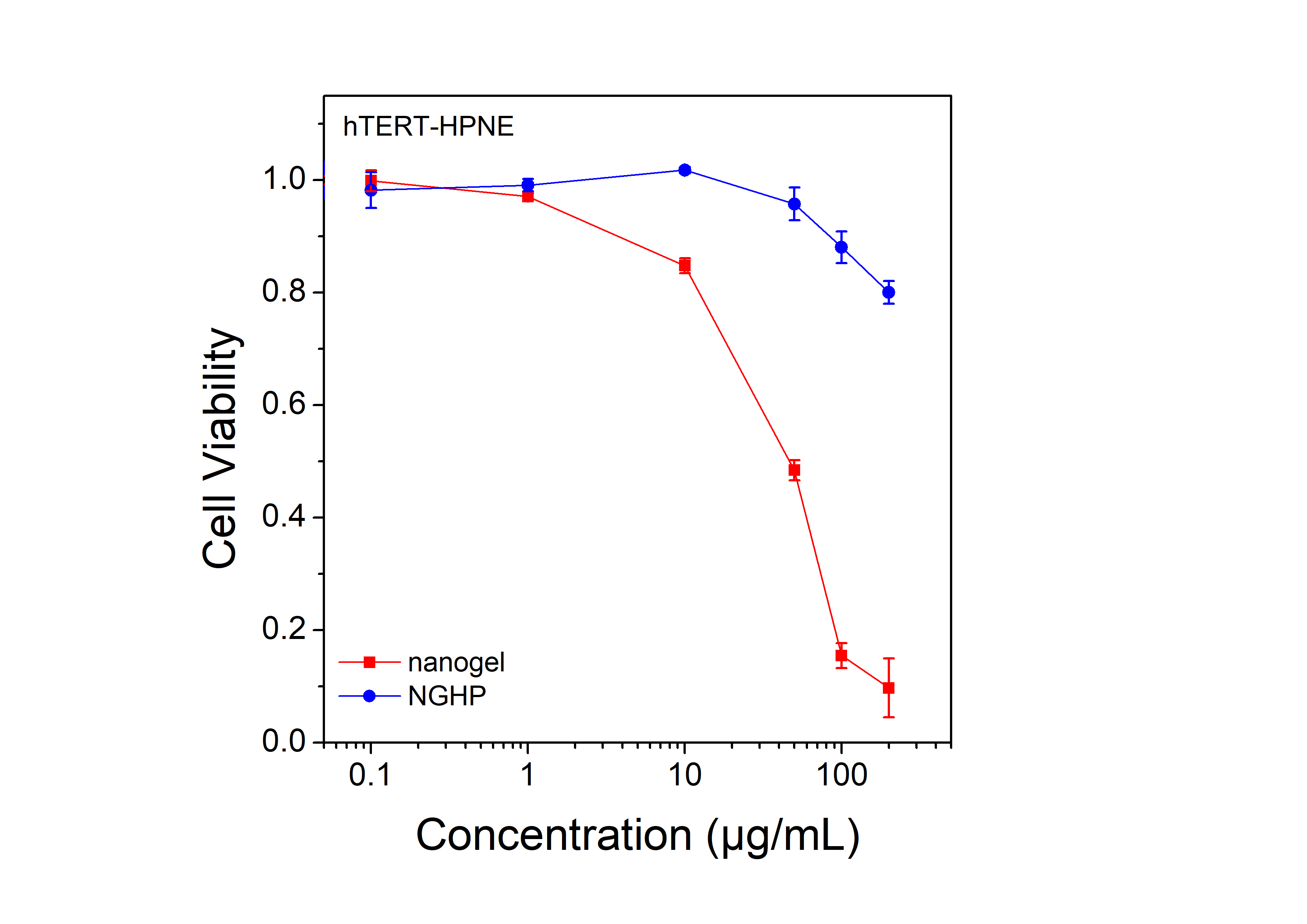
**

**Supplementary Figure 8:** Calibration curve of Abs for enzymic activity was obtained from HAase solutions. The turbid solution of blank group was used to zero the detection. The regression equation was found to be y = 0.216-0.858x with a 0.99 correlation coefficient.

**
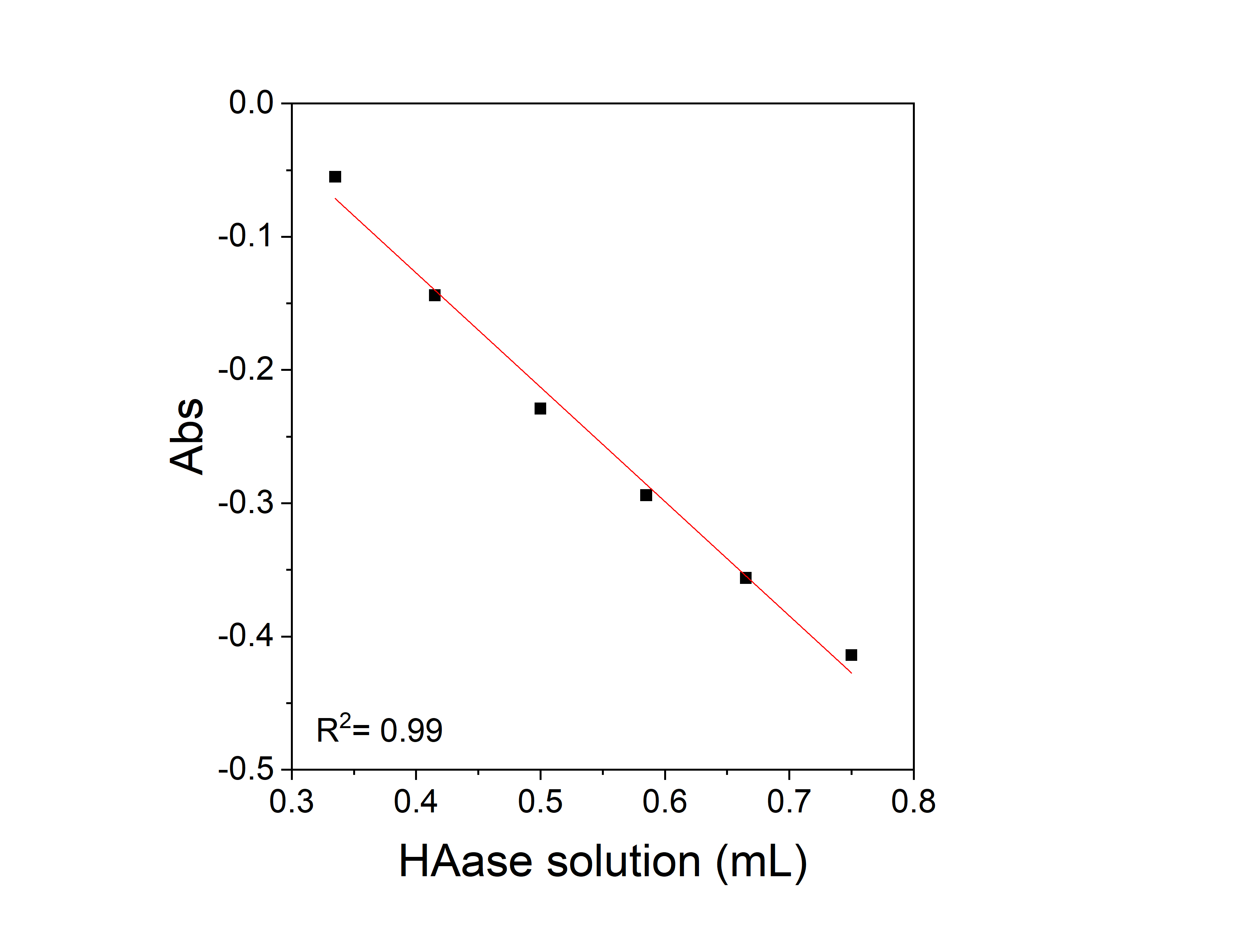
**

**Supplementary Figure 9:** (A) The proliferation inhibition by radiation on four kinds of cells. Pancreatic cancer cells were more susceptible in radiation compared to pancreatic stellate cells. (B), (C), (D) and (E) exhibited the proliferation inhibition on PANC-1, hPSC, PAN02 and mPSC by combination of PFD and radiotherapy, respectively. All data are exhibited as the mean ± SD (n = 3).


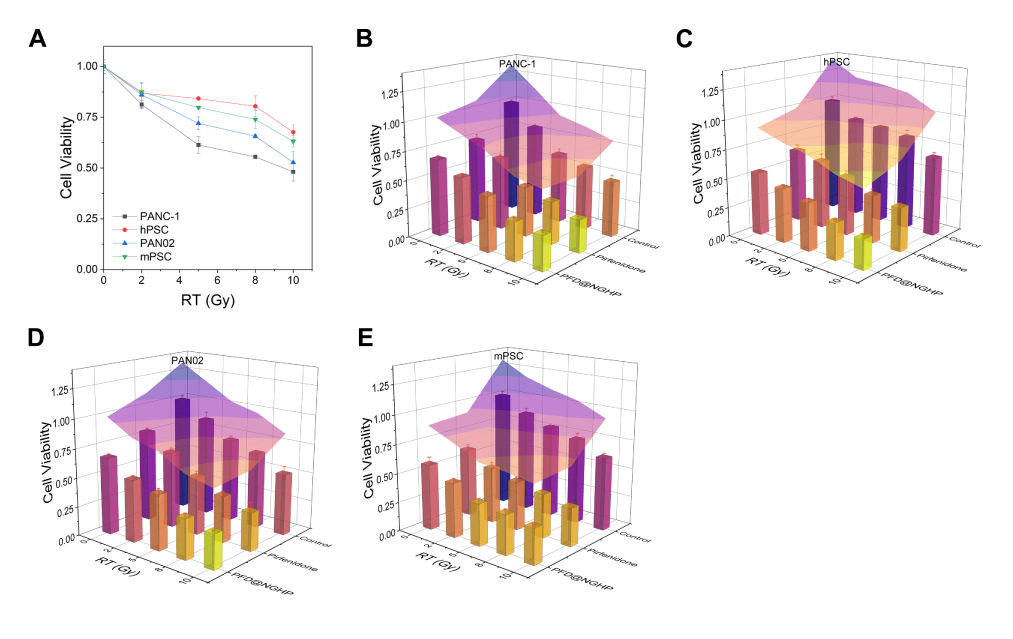


**Supplementary Figure 10:** (A) and (B): Microtumor seeds were formed by co-culture system of PANC-1/hPSC and PAN02/mPSC, respectively. Cancer cells were expressing EGFP with green fluorescence. After merging with the bright-field, cells without green fluorescence are pancreatic stellate cells. (scale bar: 500 μm). (C) and (D): The expression of α-SMA protein is a characteristic of PSCs. It can be observed that both hPSCs and mPSCs exhibit high levels of α-SMA expression. (scale bar: 20 μm).


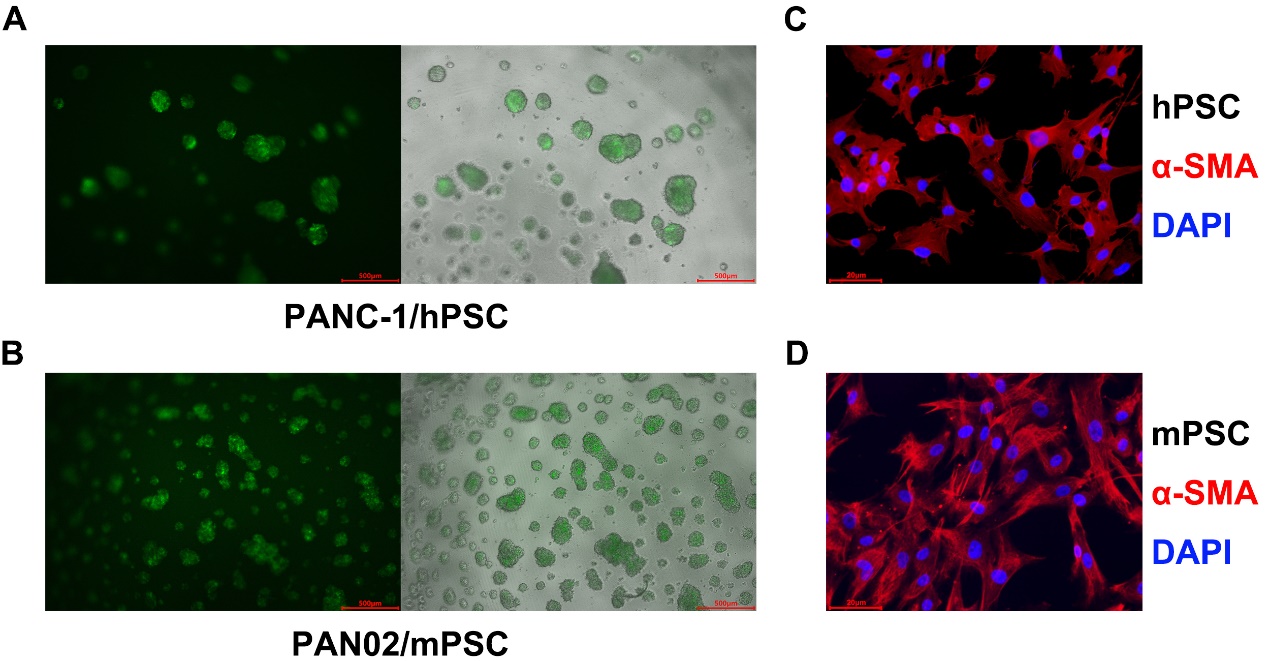


**Supplementary Figure 11: Colony formation of organotypic 3D microtumors analysed based on** **spheroids count, volume and fluorescence area ratio.**

(A) Quantitative analysis of spheroid counts obtained from Figure 6B revealed the spheroid formation capability of PANC-1&hPSC microtumors after different treatments. Spheroids with short diameter > 200um were be counted. (B) Quantitative analysis of spheroid volumes obtained from Figure 6B revealed the spheroid formation capability of PANC-1&hPSC microtumors after different treatments. Spheroid volume was estimated using the formula: Volume = Length × Width× Width / 2. (C) Quantitative analysis of fluorescence area ratio (R/G: Red fluorescence area/ Green fluorescence area) obtained from Figure 6D revealed the cellular composition in PANC-1&hPSC microtumors after different treatments. (D) Quantitative analysis of spheroid counts obtained from Figure 6C revealed the spheroid formation capability of PAN02&mPSC microtumors after different treatments. (E) Quantitative analysis of spheroid volumes obtained from Figure 6C revealed the spheroid formation capability of PAN02&mPSC microtumors after different treatments. (F) Quantitative analysis of fluorescence area ratio (R/G: Red fluorescence area/ Green fluorescence area) obtained from Figure 6E revealed the cellular composition in PAN02&mPSC microtumors after different treatments. All data are exhibited as the mean ± SD (n = 3 in A, C, D, and F. n = “spheroid count” in B and E), and the inserted asterisks indicate statistically significant differences based on *p* < 0.05(*), *p* < 0.01(**) and *p* < 0.001(***).


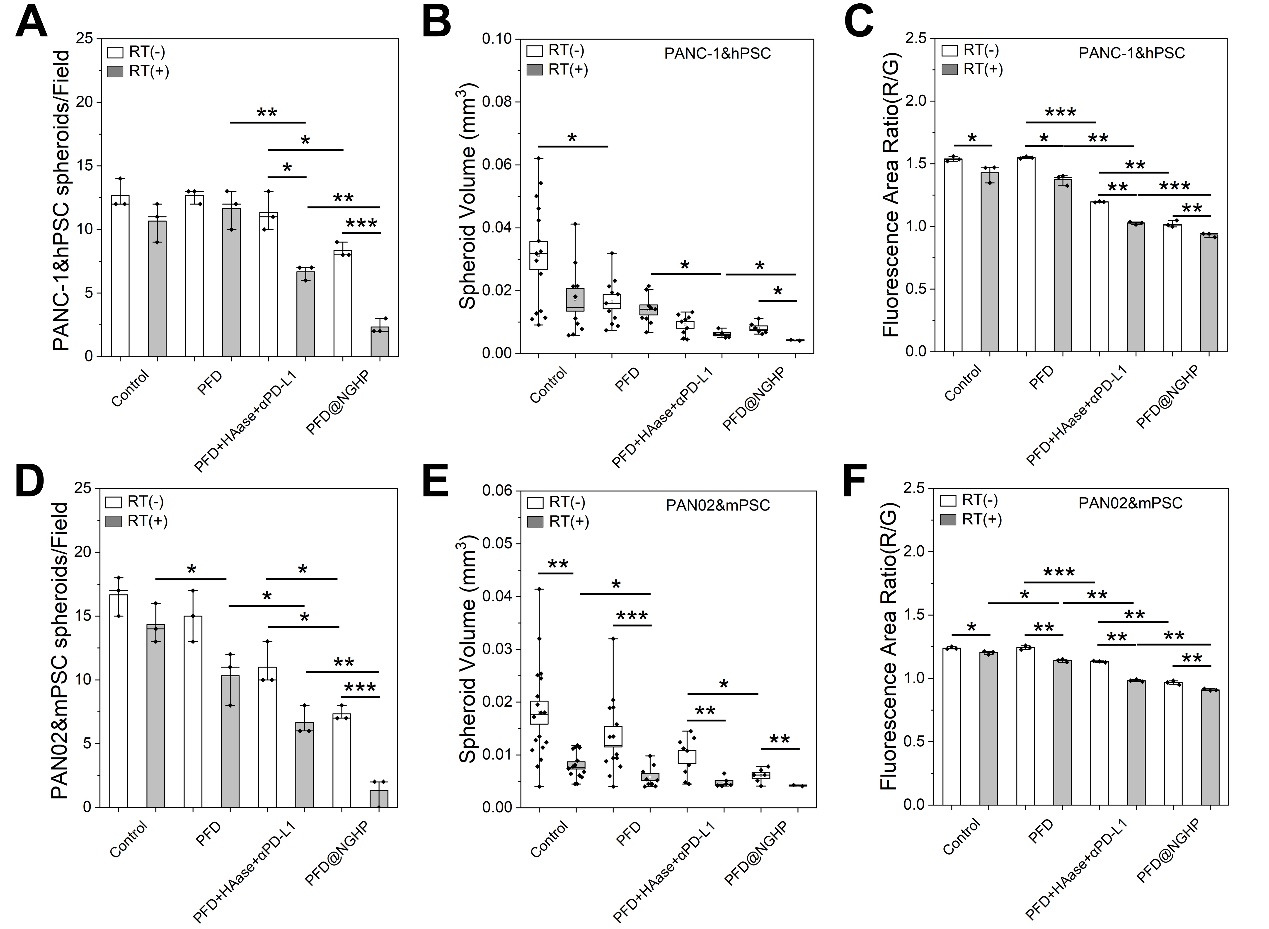


**Supplementary Figure 12:** Hematoxylin and eosin (HE) staining of vital organs from mice administered various treatments. Scale bars are 100 μm


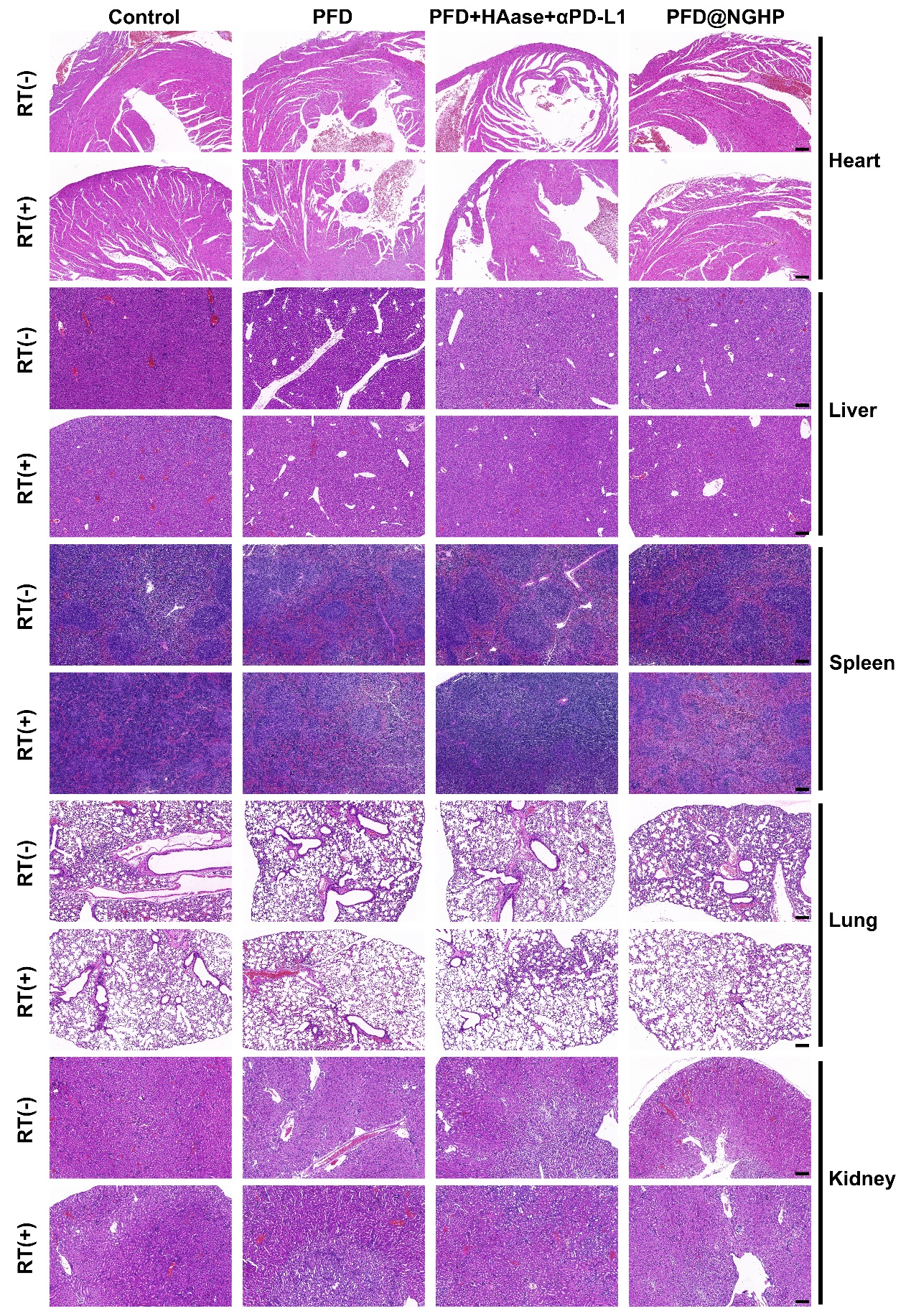


**Supplementary Figure 13:** (A) Immunofluorescence images exhibited TGF-β1 expression in non-irradiated (RT-) and irradiated (RT+) PAN02&mPSC tumors after different treatments. Red fluorescence indicated TGF-β1 and blue fluorescence indicated cellular nuclei (scale bar: 100 μm). (B)Immunohistochemistry evaluation for collagen I in tumor sections of eight groups. Collagen I was stained brown and nuclei was stained blue. Scale bars are 100 μm.


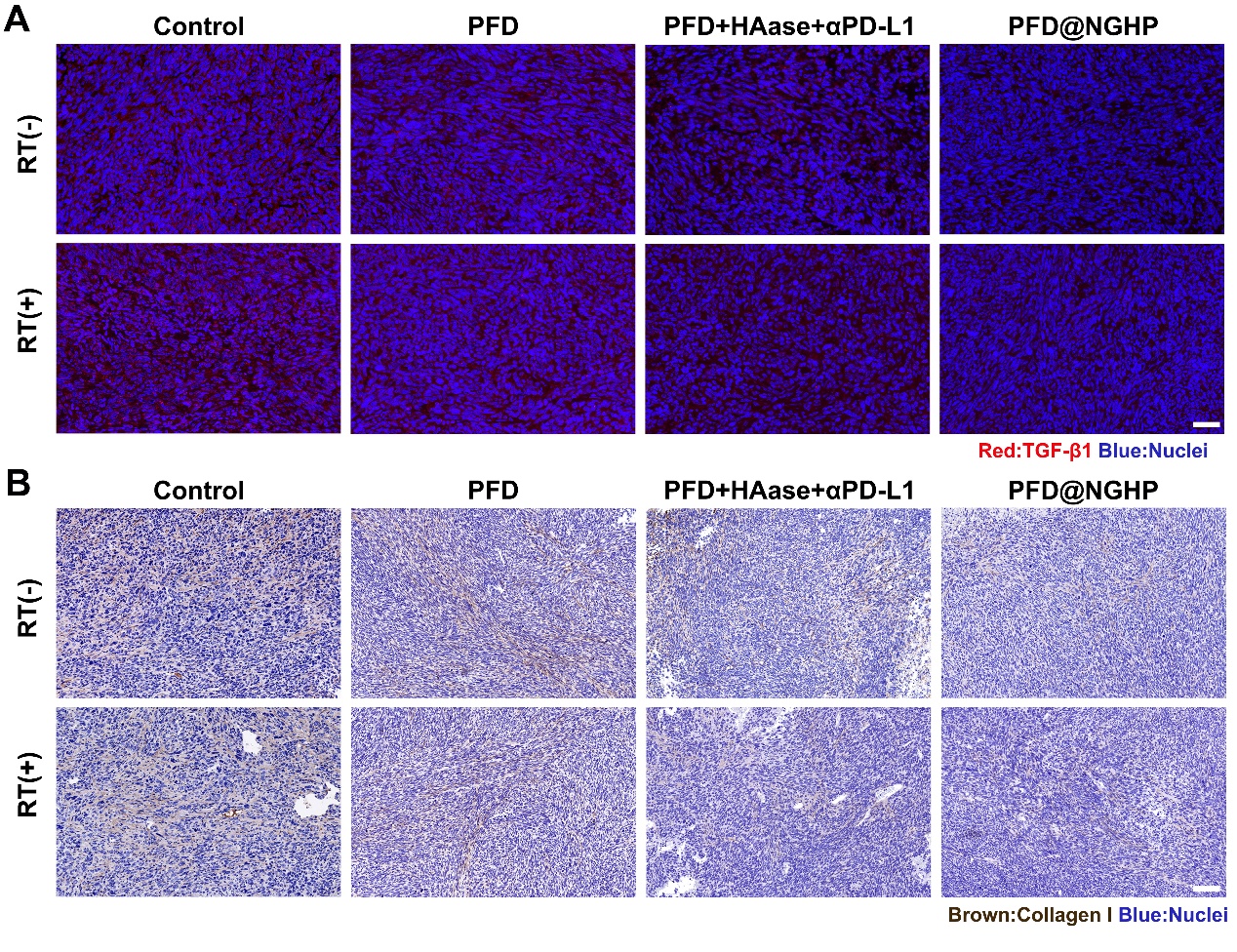


**Supplementary Figure 14:** **The area fraction representing several characteristic biomarker expressions in tumor tissues.**

(A) The area fraction of α-SMA expression obtained through semi-quantitative analysis by IHC images of Figure 7E. (B) The area fraction of hyaluronic acid (HA) expression obtained through semi-quantitative analysis by IHC images of Figure 7F. (C) The area fraction of TGF-β1 expression obtained through semi-quantitative analysis by immunofluorescence images of Supplementary Figure 13A. (D) The area fraction of collagen I expression obtained through semi-quantitative analysis by IHC of Supplementary Figure 13B. All data are exhibited as the mean ± SD (n = 3), and the inserted asterisks indicate statistically significant differences based on *p* < 0.05(*), *p* < 0.01(**) and *p* < 0.001(***).


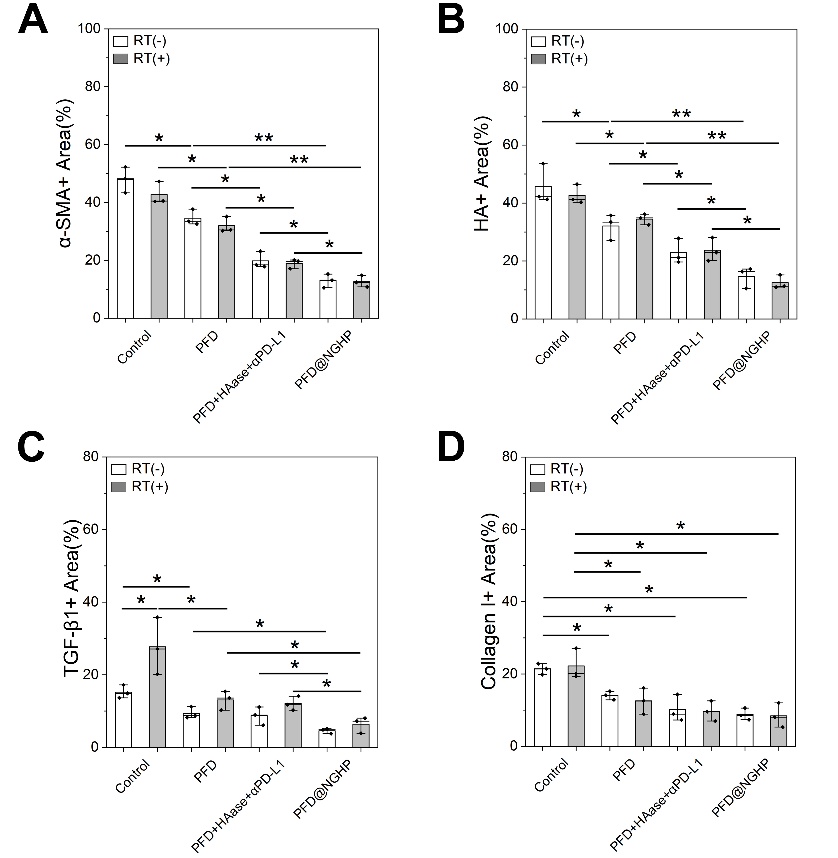


**Supplementary Figure 15: The gating strategy for CD4+/CD8+ T-cell quantification by flow cytometry**

The initial gating strategy of Figure 8B starting with Gate 1 to select the events acquired with stable flow stream, Gate 2 to select single cells, Gate 3 to select live cells (FVS780 negative), and Gate 4 to select live CD3 positive cells. Finally, CD8 positive subsets and CD4 positive subsets were sorted from CD3 positive T cells in Gate 5.


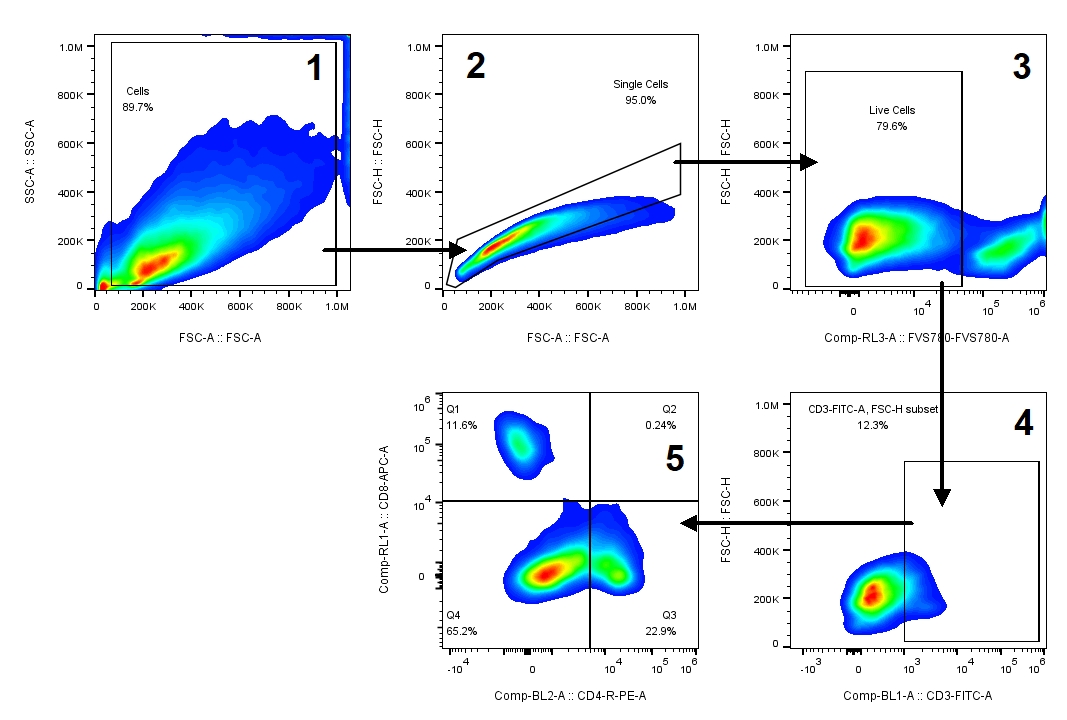


**Supplementary Figure 16: Semi-quantitative analysis of changes in immune microenvironment of tumors after different treatments.**

(A) The area fraction of PD-L1 expression in tumor tissues obtained through semi-quantitative analysis by immunofluorescence images of Figure 8A. (B) The CD4+ T cell distribution obtained through semi-quantitative analysis by immunofluorescence images of Figure 8C. (C) The CD8+ T cell distribution obtained through semi-quantitative analysis by immunofluorescence images of Figure 8C. All data are exhibited as the mean ± SD (n = 3), and the inserted asterisks indicate statistically significant differences based on *p* < 0.05(*), *p* < 0.01(**) and *p* < 0.001(***).


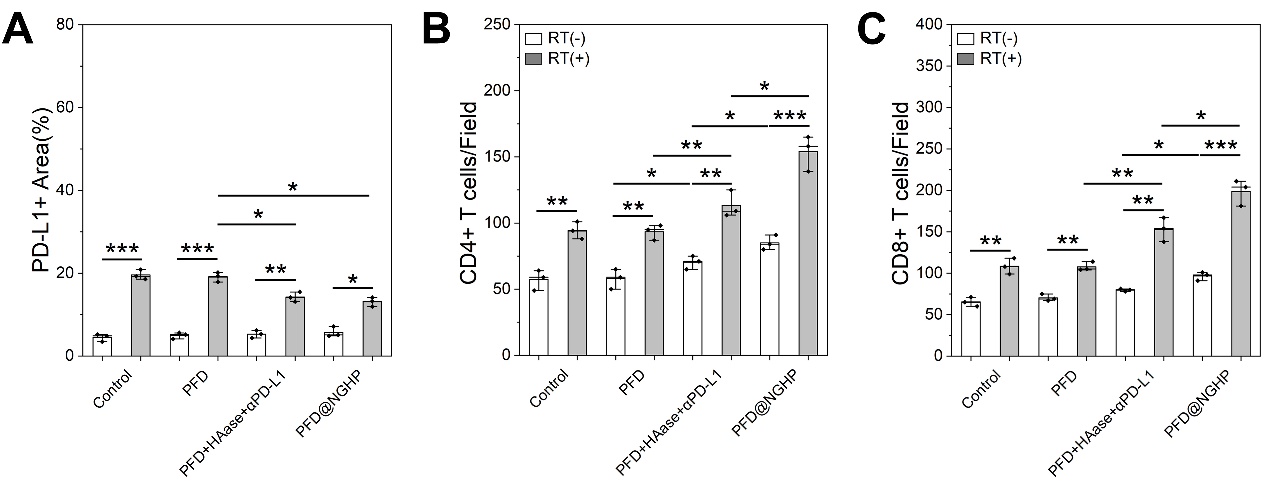


**Supplementary Figure 17: Representative images of tumor bearing mice and harvested bilateral tumors after different treatments in Figure 8D**


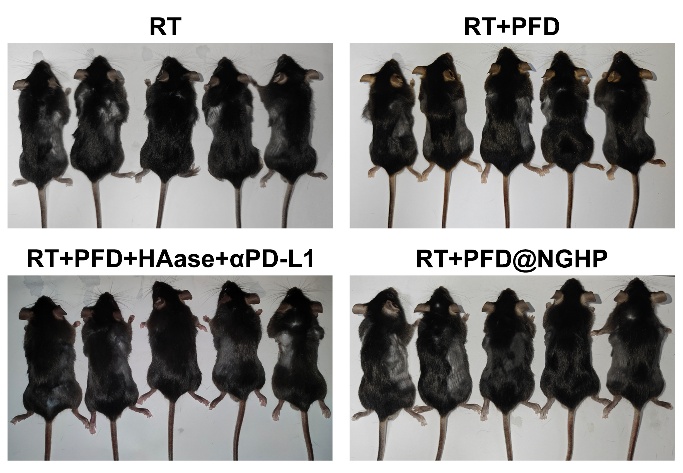

Supplement: Supplementary 1 — Figs. S1 to S17 [file bmr.0335.f1.docx]
